# Competitive behaviors in *Serratia marcescens* are coordinately regulated by a lifestyle switch frequently inactivated in the clinical environment

**DOI:** 10.1016/j.chom.2025.01.001

**Published:** 2025-01-29

**Authors:** David J. Williams, Alexandra Hawkins, Ruth E. Hernandez, Giuseppina Mariano, Katharine Mathers, Grant Buchanan, Barnaby J. Stonier, Teresa Inkster, Alistair Leanord, James D. Chalmers, Nicholas R. Thomson, Matthew T.G. Holden, Sarah J. Coulthurst

**Affiliations:** 1 School of Life Sciences, University of Dundee, Dundee DD1 5EH, UK; 2 Wellcome Sanger Institute, Hinxton CB10 1SA, UK; 3 Antimicrobial Resistance and Healthcare Associated Infection (ARHAI), Glasgow, Scotland; 4 School of Infection and Immunity, University of Glasgow, Glasgow G12 8TA, UK; 5 Scottish Microbiology Reference Laboratories, Glasgow G31 2ER, UK; 6 School of Medicine, University of Dundee, Dundee DD1 9SY, UK; 7 London School of Hygiene & Tropical Medicine, London WC1E 7HT, UK; 8 School of Medicine, University of St Andrews, St. Andrews KY16 9TF, UK

## Abstract

Opportunistic bacterial pathogens must compete with other bacteria and switch between host- and environment-adapted states. Type VI secretion systems (T6SSs) occur widely in gram-negative bacteria and can efficiently kill neighboring competitors. We determined the distribution of T6SSs across the genus *Serratia* and observed that a highly conserved antibacterial T6SS is differentially active between closely related clinical isolates of *Serratia marcescens*. By combining genomic and experimental approaches, we identified a genus-core two-component system, BetR-Reg1-Reg2, that controls T6SS activity and exhibits frequent inactivating mutations, exclusively in *S. marcescens* isolates of clinical origin. This regulatory system controls a number of lifestyle-related traits at transcriptional and post-translational levels, including T6SS activity, antibiotic production, motility, and adhesion, with loss of BetR increasing virulence in an *in vivo* infection model. Our data support a model whereby this system represents a conserved, modular switch from sessile to pioneering and aggressive behavior, which is subject to selection pressure in clinical environments.

## Graphical abstract

**Graphic abstract:**
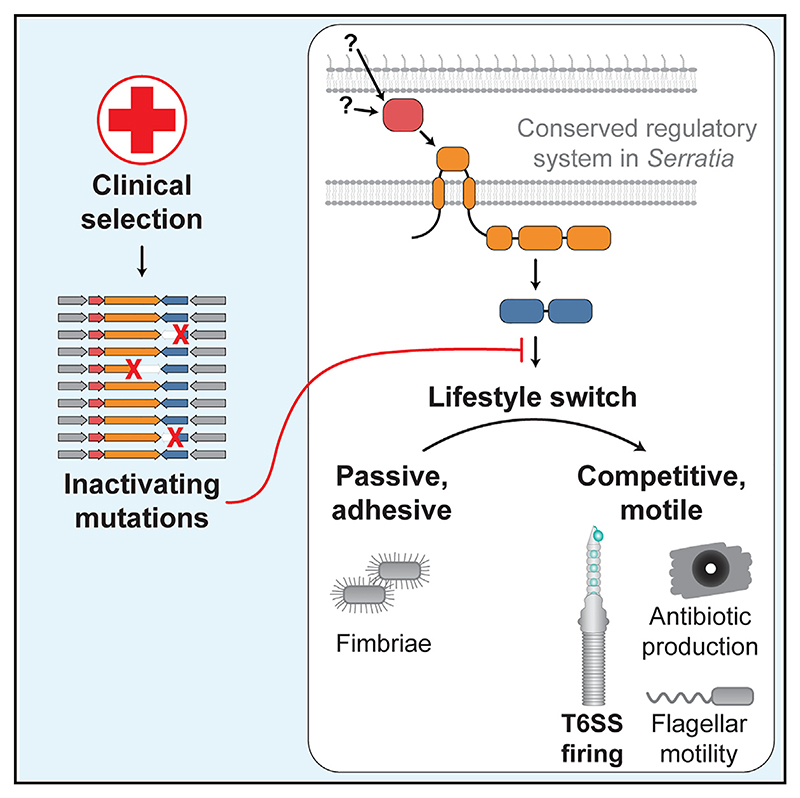

## Introduction

Bacteria can respond to, adapt to, and actively change their environment to occupy a myriad of niches, including to promote colonization and subsequent survival within a host. Given that bacteria typically exist within polymicrobial communities, both in the environment and in association with host organisms, a key aspect of surviving and manipulating their environment is the ability to compete with neighboring microbes for space and nutrients.^1^ Inter-bacterial competition is ubiquitous and includes directly attacking rivals by releasing diffusible antibiotics and contact-dependent delivery of protein toxins into neighboring cells.^2^ The type VI secretion system (T6SS) occurs widely in gram-negative bacteria and is used to deliver toxic effector proteins directly into adjacent cells. It is a large, contractile machinery that fires a spear-like puncturing structure carrying multiple effector proteins into target cells.^3^ The T6SS is highly versatile and can be used against bacteria, higher eukaryotes, fungal cells, and to scavenge metal ions.^4^ However, its primary function is antibacterial, being used to efficiently kill or disable competitor bacteria by intoxicating them with diverse antibacterial effectors.^5^ Growing evidence suggests that T6SS-mediated antagonistic inter-bacterial interactions play a central role in shaping beneficial host-associated microbial communities, including the human gut microbiota, and can promote pathogen invasion of the microbiota and hence colonization or infection in several animal models.^6,7^ Importantly, however, direct evidence for the role and impact, positive or negative, of antibacterial T6SSs deployed by bacterial pathogens during human infection remains very limited.

*Serratia marcescens* is an opportunistic pathogen that is ubiquitous in the environment and represents a significant cause of antibiotic-resistant hospital-acquired infections, typically in immunocompromised patients.^8,9^ The model strain Db10 possesses an antibacterial T6SS that is transcriptionally and post-translationally regulated to fire constitutively under normal conditions.^10,11^ This strain also produces althiomycin, a broad-spectrum antibiotic synthesized by a nonribosomal peptide synthetase-polyketide synthase (NRPS-PKS), while genes encoding many other known or putative antimicrobial specialized metabolites are found across the genus *Serratia*.^12,13^ However, Db10 was isolated from an insect and is not representative of *S. marcescens* associated with human carriage or disease. Recent large-scale genomic studies of *Serratia* and *S. marcescens* have revealed that *S. marcescens* is a complex species separated into distinct lineages, including several highly associated with clinical origin,^9,13,14^ but the distribution of T6SSs in *Serratia* has not been examined in the context of such a phylogenomic framework. Moreover, not only is the potential impact of antibacterial T6SSs during clinical infections by *S. marcescens* unexplored, we lack a broader understanding of how this organism evolves to adapt to clinical environments.

Here, we report that while the Db10-like T6SS is conserved across *Serratia*, we observe marked differences in T6SS activity between very closely related clinical isolates, resulting from multiple independent mutations in a three-gene regulatory locus. This conserved regulatory system positively regulates T6SS activity but also controls a broader set of lifestyle-associated genes in *S. marcescens* and is subject to frequent inactivating mutations, exclusively in clinical isolates. Our results indicate that the T6SS is co-regulated with other competitive traits as part of a conserved lifestyle switch that is subject to strong selection pressure associated with the clinical environment.

## Results

### The T6SS of *Serratia marcescens* Db10 is conserved across multiple species of *Serratia*

**Figure 1:**
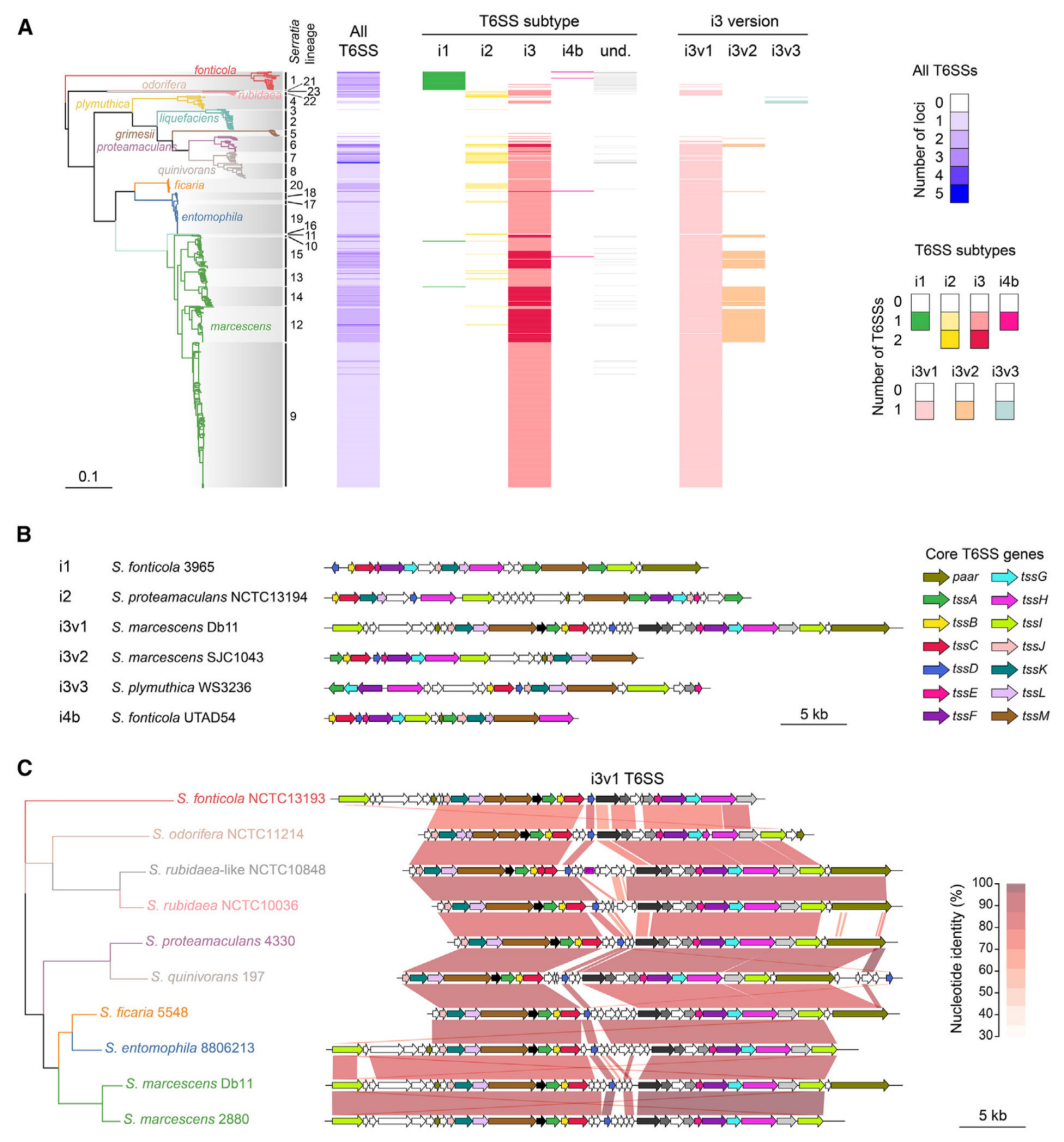
T6SSs across the genus *Serratia* (A) Maximum-likelihood phylogenetic tree of 664 *Serratia* genomes from a core-gene alignment (determined previously^13^) with corresponding presence/absence of T6SS-encoding gene clusters in each strain. T6SS subtypes were classified through phylogenetic analysis of a concatenated TssB-TssC sequence alignment. Clades are colored according to *Serratia* species, and alternating shaded boxes distinguish lineages. Columns adjacent to the tree show the presence of (from left to right): all T6SS loci identified; then T6SSs of subtypes i1, i2, i3, i4b, and undetermined (und); and then i3 subtypes separated into i3v1, i3v2, and i3v3. The strength of the color represents the number of T6SSs identified in each grouping. Undetermined T6SSs were either outgrouped from the reference T6SS dataset or full-length TssB/TssC sequences were not identified. (B) Examples of gene clusters representing the different T6SS subtypes (i1-i4b) identified in *Serratia*. Genes encoding core T6SS components are colored according to the key. Genomic identifiers are given in Figure S1. (C) Synteny plot of representative i3v1 T6SS gene clusters from each species of *Serratia* that encodes the i3v3 subtype, alongside a representative phylogenetic tree showing the position of each corresponding strain, extracted from the tree above. Shading between representative gene clusters indicates pairwise percentage nucleotide identity. *S. marcescens* Db11 is a single nucleotide variant derivative of strain Db10; therefore, the Db11 genome sequence is used interchangeably for Db10 and Db11. See also Figures S1 and S7 and Table S1.

In order to determine the occurrence and distribution of the T6SS in *Serratia*, we searched for T6SSs within a comprehensive set of 664 genomes spanning the genus.^13^ This set includes clinical and environmental isolates and represents 23 defined lineages, including seven within the species *marcescens*^13^ (Figure 1A). Multiple T6SSs are found in *Serratia*, including examples of i1, i2, i3, and i4 subtypes (Figures 1A, 1B, and S1A; Table S1). There are three distinct i3 T6SSs, including the model T6SS from *S. marcescens* Db10, which is classified here as T6SS_i3v1. T6SS_i3v1 is present across *Serratia* in a paraphyletic pattern (and is also carried by two *Serratia fonticola* genomes). A second i3 T6SS, T6SS_i3v2, with similarity to the metal-scavenging T6SS-4 in *Burkholderia* and *Yersinia*,^15,16^ is present in several lineages of *S. marcescens* in a polyphyletic pattern. T6SS_i3v1 is highly conserved in most *Serratia* species, and a synteny comparison of regions flanking T6SS_i3v1 in representative genomes shows that this T6SS is encoded in the same chromosomal locus in each of these species, except for the two *S. fonticola* genomes (Figure S1B). In this common location, the region upstream of T6SS_i3v1 is conserved while the region downstream is more diverse. Given the position of the most recent common ancestor of genomes encoding T6SS_i3v1 (Figure 1A), parsimony suggests that T6SS_i3v1 has been lost in *Serratia liquefaciens, Serratia plymuthica*, and *Serratia grimesii* on independent occasions, supported by differences in the remaining genes at the site of loss (Figure S1C). Within the T6SS_i3v1 gene clusters, consistent with previous observations,^17^ genes encoding core T6SS components are conserved between species and strains, but regions encoding effector proteins, here around *tssD* (*hcp1*), are highly variable, indicating horizontal exchange of T6SS effectors (Figure 1C).

### Closely related clinical isolates of *Serratia marcescens* display distinct T6SS phenotypes

**Figure 2:**
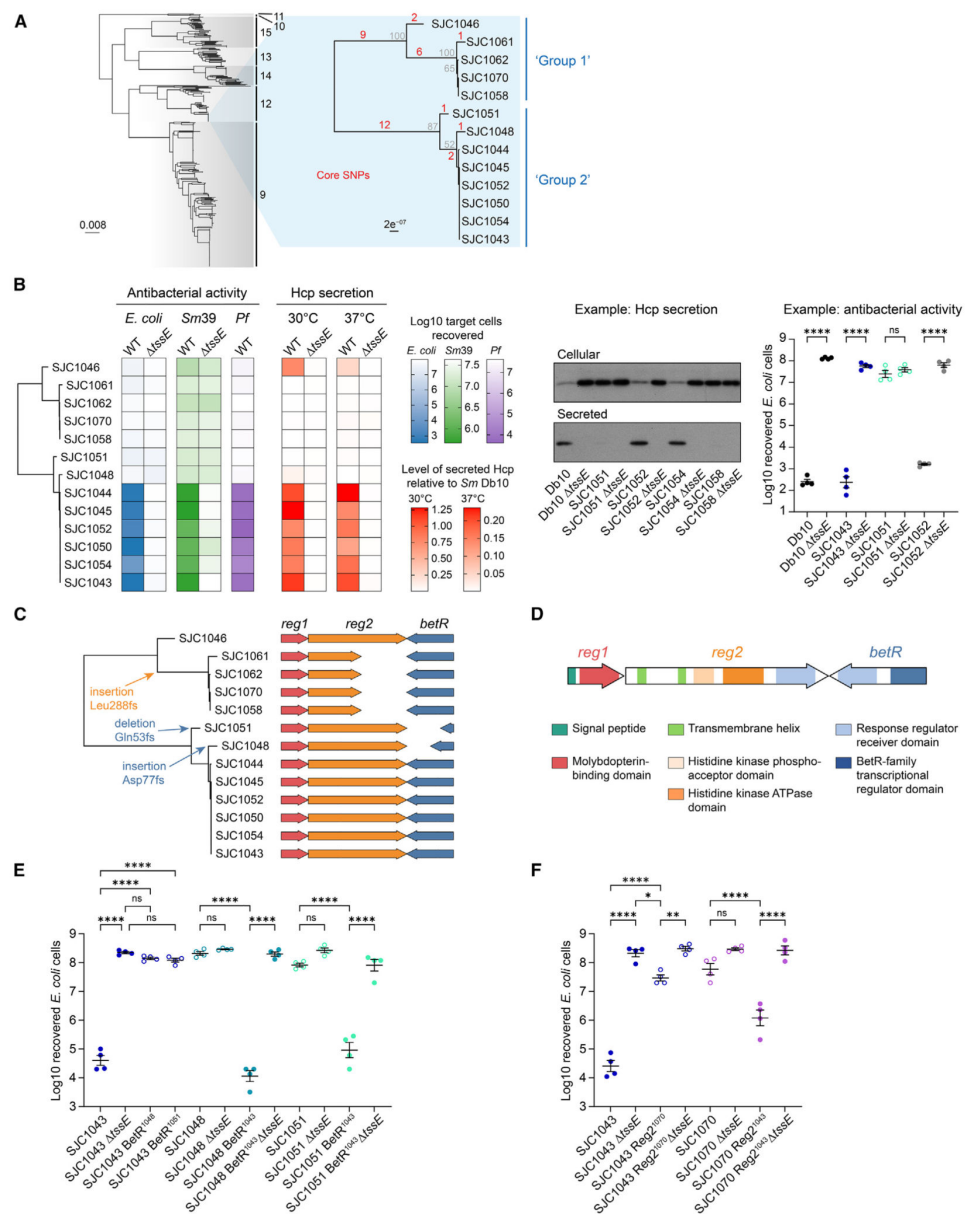
Independent mutations in a two-component regulatory system lead to loss of T6SS-dependent antibacterial activity in clinical isolates of *Serratia marcescens* (A) Phylogenetic tree of 404 *S. marcescens* genomes (extracted from Figure 1A). Inset: maximum-likelihood phylogenetic tree of a group of closely related clinically derived isolates, based on SNP alignment (reads mapped to isolate SJC1043). Bootstrap values are in gray, core SNP distances in red. (B) Phylogenetic tree of clinical isolates (part A, inset) alongside columns representing (from left to right): antibacterial activity of wild-type (WT) and T6SS-inactive (Δ*tssE*) mutants of each clinical *S. marcescens* isolate against *E. coli* and *S. marcescens* SM39 target organisms, antibacterial activity of wild-type isolates against a *Pseudomonas fluorescens* (*Pf*) target, and Hcp secretion by wild-type and Δ*tssE* strains at 30°C and 37°C. For antibacterial activity, the number of recovered target cells following co-culture with the indicated strain of *S. marcescens* is plotted (mean of four replicates). For Hcp secretion, the amount of secreted Hcp determined by immunoblotting is plotted (mean of two replicates). The darker the color, the higher the level of antibacterial activity (i.e., the lower the number of recovered target cells) or the higher the level of Hcp secretion. Examples of anti-Hcp immunoblots of cellular and secreted fractions and individual co-culture assays are shown on the right, and the full data are in Figure S2. (C) To-scale representation of the *betR*-containing locus, comprising genes *reg1* (red), *reg2* (orange), and *betR* (blue), in each isolate. Amino acid changes caused by frameshift mutations in *betR* and *reg2* are highlighted in the tree at the most parsimonious locations. (D) Predicted domains in the proteins encoded by the *betR, reg1*, and *reg2* genes. (E and F) Recovery of target *E. coli* cells following co-culture with wild-type clinical isolates or derivatives with an exchanged *betR* (E) or *reg2* (F) allele. SJC1043 BetR^1048^ indicates SJC1043 with the *betR* allele from SJC1048, and similarly for the other exchanges. Data from co-culture assays are presented as mean ± SEM with individual data points overlaid (*n* = 4 biological replicates; ****p < 0.0001, **p < 0.01, *p < 0.05, ns not significant; one-way ANOVA with Tukey’s test; for clarity, only selected comparisons are displayed). Filled circles indicate isolates with *betR* and *reg2* intact, and open circles indicate isolates with *betR* or *reg2* disrupted. See also Figures S2 and S3.

Having identified T6SS_i3v1 in clinically associated lineages of *S. marcescens* (9, 12, and 14; Figure 1), we determined whether such clinical isolates display T6SS-dependent antibacterial activity. Of particular interest was a group of 13 very closely related isolates in lineage 12 that were isolated from a single UK hospital within a 2-year period. These isolates are separated into two phylogenetic groups, group 1 (maximum nine core SNPs) and group 2 (maximum four core SNPs) (Figure 2A), with the two groups separated by 21 SNPs. The 13 isolates showed striking differences in their ability to outcompete other species in a T6SS-dependent manner, as measured by recovery of “target” competitor bacteria following co-culture with wild-type or T6SS inactive (Δ*tssE*) derivatives of each clinical isolate (Figures 2B and S2). Six of the isolates in group 2 caused a large T6SS-dependent reduction in target cell recovery, while two isolates, SJC1048 and SJC1051, did not, similar to those in group 1. In all 13 isolates, Hcp, a structural T6SS protein secreted when the system fires, could be detected inside the cells. However, secreted Hcp was only detected in the isolates displaying T6SS-dependent antibacterial activity, consistent with only those isolates having an actively firing T6SS and with the difference in T6SS activity operating at a post-translational level (Figures 2B and S4). One exception is isolate SJC1046, the most genetically divergent member of group 1 (distinguished from the rest of group 1 by eight core SNPs), which showed modest Hcp secretion but little antibacterial activity. These 13 isolates also encode T6SS_i3v2 (Figures 1A and 1B), but we have not been able to detect activity of this system.

### Independently occurring mutations in a two-component regulatory system lead to loss of T6SS activity in clinical isolates

The divergent T6SS phenotypes observed between the closely related clinical isolates do not correlate with large chromosomal variations or differences in plasmid carriage (Figures S3A and S3B). However, they do correlate with three independently occurring frameshift mutations in the same genetic locus, which each lead to premature termination and predicted loss of function of the protein products (Figure 2C). This locus contains three genes encoding a predicted two-component phosphorelay system, namely genes encoding a possible periplasmic sensing protein (regulator 1 [*reg1*]), a transmembrane histidine kinase (regulator 2 [*reg2*]), and a DNA-binding BetR-family response regulator (*betR*) (Figure 2D).

In order to confirm that the mutations in *betR* and *reg2* were the cause of the loss of T6SS activity, we precisely exchanged intact and mutation-containing *reg2* and *betR* genes between individual isolates and assessed T6SS-dependent antibacterial activity against *E. coli* and *Pseudomonas fluorescens* (Figures 2E, 2F, and S2). Introduction of either of the non-identical frameshifted *betR* sequences from SJC1048 and SJC1051 in place of intact *betR* in the T6SS-active isolate SJC1043 resulted in little or no T6SS activity, comparable with SJC1048 and SJC1051. Inversely, repair of the frameshifted *betR* genes in SJC1048 and SJC1051 to the intact *betR* sequence from SJC1043 resulted in restoration of antibacterial activity, dependent on a functional T6SS (Figures 2E and S2E). Similarly, introduction of the frameshifted *reg2* allele from SJC1070 into SJC1043 resulted in a marked reduction in T6SS-dependent antibacterial activity, to the same level as SJC1070. In this case, replacement of the mutated *reg2* allele in SJC1070 with the intact version from SJC1043 restored an intermediate level of T6SS activity against *E. coli* (Figure 2F) but not *P. fluorescens* (Figure S2F). This lack of substantial antibacterial activity of Reg2-restored SJC1070 against *P. fluorescens* is reminiscent of the phenotype of SJC1046, the group 1 strain that has an intact *reg2* gene. We also determined that mutation of *betR* or *reg2* did not have a significant impact on growth rate (Figure S3C). Taken together, the data show that intact *betR* and *reg2* genes are required for efficient T6SS activity but may not be sufficient for maximal activity in group 1-like isolates of *S. marcescens*.

### Loss-of-function mutations in *betR* and *reg2* are found frequently and exclusively in clinically derived *Serratia marcescens*

**Figure 3:**
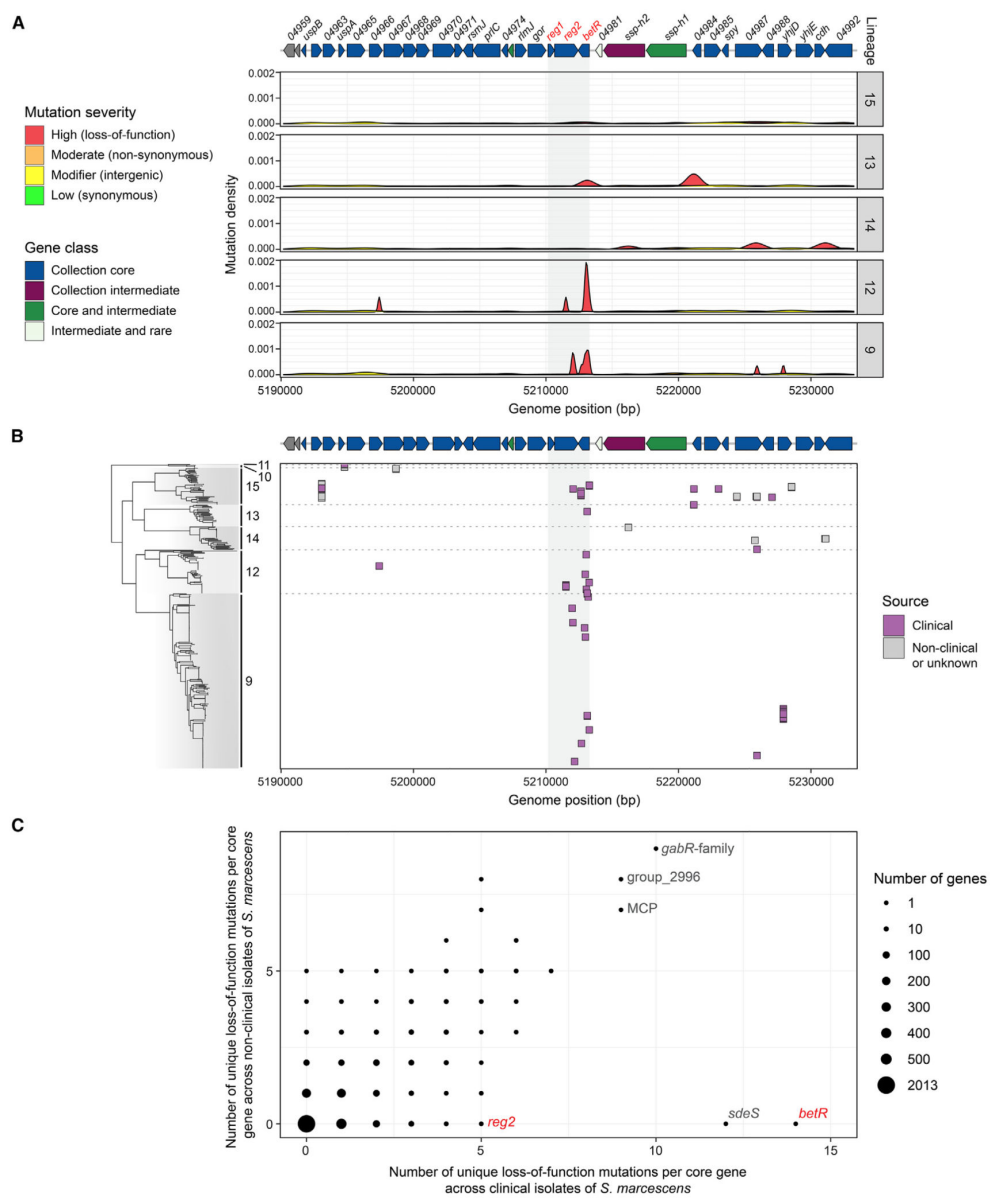
Loss-of-function mutations in the *betR* locus occur frequently and exclusively in clinically derived isolates of *Serratia marcescens* (A) Density plot of mutations in the *betR* locus and 40 kb flanking regions, compared with SJC1043, across *S. marcescens*. Mutations are categorized by severity (defined using SnpEff): high—frameshift, loss of start codon, loss or gain of stop codon (loss of function, red); moderate—single amino acid change (non-synonymous, orange); modifier—in intergenic region (yellow); low—no amino acid change (synonymous, green). Density plots are shown for the main lineages in *S. marcescens*, ordered from top to bottom as in the phylogenetic tree in (B). Above the plot is a to-scale representation of the genes in this region, with the *betR* locus highlighted by red labels and genes colored by level of conservation across *marcescens* in a lineage-weighted manner (gene class, assigned using Twilight^18^). (B) Individual loss-of-function mutations shown in relation to the position of the isolate in the *S. marcescens* phylogeny. Mutations in clinically derived isolates are shown in lilac, and those in non-clinically derived isolates (or isolates of unknown origin) are in gray. (C) Number of unique loss-of-function (high severity) mutations per gene observed in *S. marcescens* isolates from clinical environments vs. number of unique loss-of-function mutations per gene in isolates from non-clinical or unknown environments. The size of the dot indicates the number of core genes with the corresponding number of mutations. Mutations were determined using SJC1043 as the reference, and each unique mutation was counted only once in either environment to remove bias from mutations fixed in other lineages or in clonal isolates that are over-represented in the dataset. See also Figure S4 and Data S1.

The appearance of three separate frameshift mutations within two genes in the *betR* locus in these closely related isolates is very striking, particularly given that the mutation rate for *S. marcescens* is estimated to be one to two nucleotide substitutions per genome per year.^9^ Therefore, we investigated the conservation and occurrence of loss-of-function mutations in this locus further. Interrogation of a previously constructed pangenome of *Serratia*^13^ revealed that the *betR* locus is conserved across *Serratia*, and the three genes in this locus, *reg1, reg2*, and *betR*, are always encoded together as an invariant unit (Figure S4). Examination of sequence variation in the *betR* locus and flanking regions, using SJC1043 as a reference, highlighted that loss-of-function variants, such as those causing loss of a start codon or generation of a premature stop codon, are disproportionately found in *reg2* and *betR* compared with the surrounding regions (Figures 3A and 3B). By contrast, non-synonymous and synonymous variants are equally distributed across the *betR* locus and surrounding regions (Figure 3A). Importantly, loss-of-function variants in *betR* and *reg2* are only seen in isolates of clinical origin (Figure 3B) and are predominantly found in the clinically associated lineages 12 and 9, which contain clonal populations of *S. marcescens* circulating in UK hospitals.^9^ These variants are not fixed in the clonal groups (Figure 3B) and may represent recent selection events.

There is a high occurrence of independent mutations within the identified loss-of-function variants in *reg2* and *betR*, with five and fourteen distinct loss-of-function variants observed in these two genes, respectively, and, as noted above, these variants were only observed in isolates of clinical origin (Figure 3C). In fact, across all core genes in *S. marcescens, betR* was found to be the gene with the highest number of unique loss-of-function variants exclusive to isolates of clinical origin (Figure 3C; Data S1), suggesting a clinically associated selection for inactivated BetR-mediated regulation in isolates recently persisting in this environment. Further potential evidence of clinical selection was found for *sdeS*, which encodes a transcriptional repressor of the SdeAB multidrug efflux pump.^19^ Similar to *betR*, a high number of unique loss-of-function mutations were observed in *sdeS*, but only in isolates of clinical origin (Figure 3C). These *sdeS* mutations presumably result in elevated efflux pump expression and resistance to antimicrobial agents in response to selection pressure from the use of such compounds in a clinical environment. Our data suggest that inactivation of the BetR regulatory pathway is also a consequence of a clinically associated selection pressure.

### BetR controls T6SS activity across multiple lineages of *Serratia marcescens*

**Figure 4:**
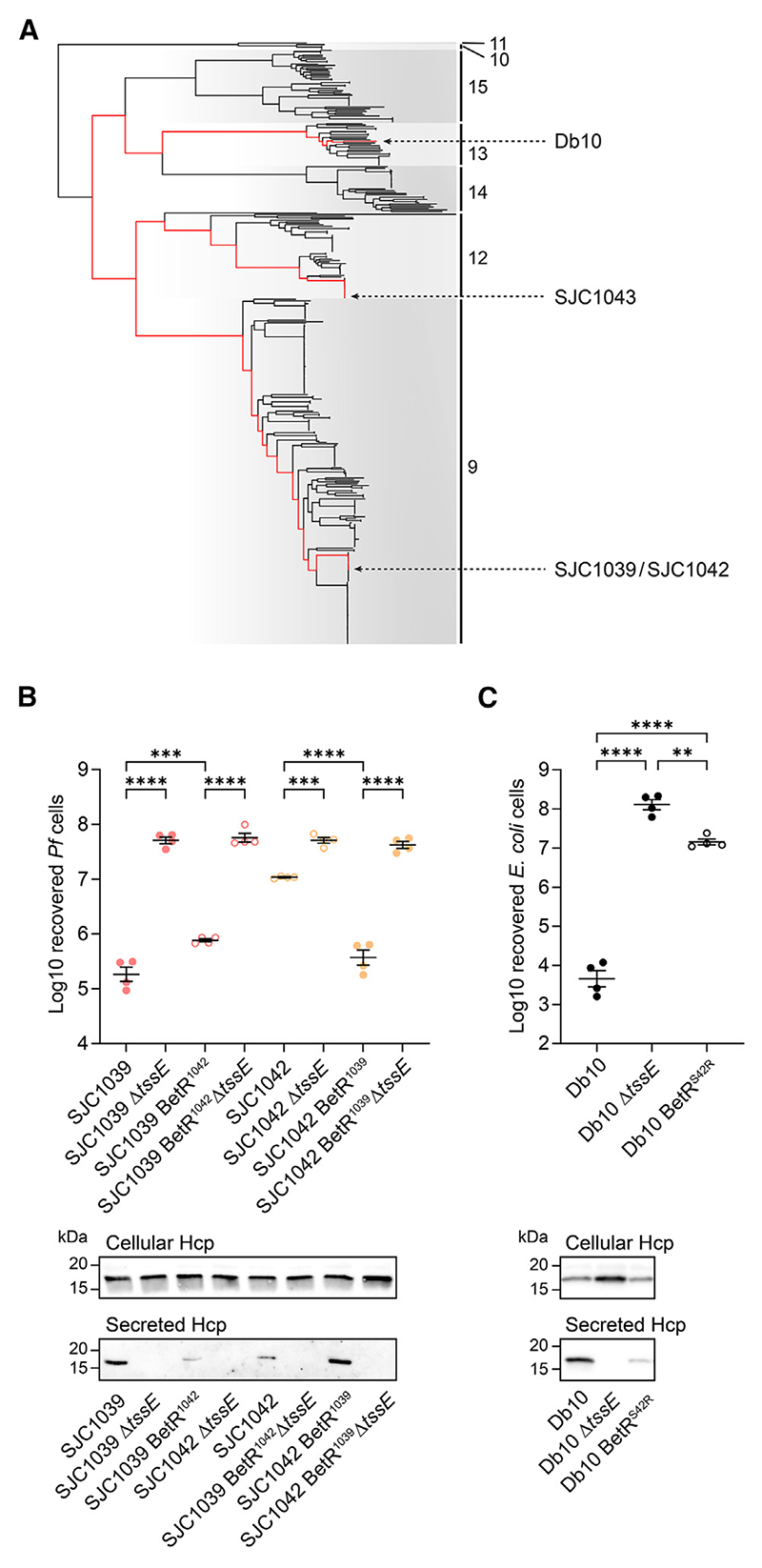
The impact of *betR* mutation on T6SS activity is observed in distinct lineages of *Serratia marcescens* (A) Phylogenetic tree of 404 *S. marcescens* genome sequences (extracted from Figure 1A) highlighting the position of isolates of interest. Red lines show the path to the most recent common ancestor for Db10, SJC1043, and SJC1039/SJC1042. (B) T6SS-dependent antibacterial activity (top) and Hcp secretion (bottom) of wild-type clinical isolates SJC1039 and SJC1042 and derivatives with an exchanged *betR* and/or inactive T6SS (Δ*tssE*). SJC1039 BetR^1042^ indicates SJC1039 with the *betR* allele from SJC1042 and vice versa. (C) T6SS-dependent antibacterial activity (top) and Hcp secretion (bottom) of wild-type Db10 and derivatives with an inactive T6SS (Δ*tssE*) or a single base pair mutation in *betR* (T126G) resulting in a Ser42Arg substitution in BetR (Db10 BetR^S42R^). In (B) and (C), antibacterial activity is presented as recovery of *P. fluorescens* (*Pf*) or *E. coli* target cells following co-culture with the indicated strains of *S. marcescens*, and Hcp secretion is shown by immunoblot detection of Hcp in cellular and supernatant fractions. Data from co-culture assays are presented as mean ± SEM with individual data points overlaid (*n*=4 biological replicates; ****p < 0.0001, ***p < 0.001, **p < 0.01; one-way ANOVA with Tukey’s test; for clarity, only selected comparisons are displayed). Filled circles indicate isolates with *betR* intact, and open circles indicate isolates where *betR* is disrupted or carrying a point mutation.

While investigating variants in *betR* and *reg2* across *S. marcescens*, we noticed that two closely related clinical isolates in our collection, SJC1039 and SJC1042, differed in their *betR* genotype. These isolates sit within a different lineage to SJC1043 and differ from each other by ∼100 SNPs (Figure 4A). While SJC1039 encodes full-length BetR, SJC1042 has a single base pair deletion in *betR*, which shifts the reading frame at the second codon. SJC1042 displays a low level of Hcp secretion and T6SS-dependent antibacterial activity, but T6SS activity could be restored to the same level as that of SJC1039 by introduction of intact *betR* from SJC1039 (Figure 4B). Conversely, replacement of the intact *betR* gene in SJC1039 with the frameshifted version from SJC1042 caused a reduction in T6SS activity. Therefore, BetR also positively regulates the T6SS in these isolates. Separately, we identified a T6SS-deficient mutant of *S. marcescens* Db10 with a spontaneous single base pair mutation resulting in a Ser42→Arg substitution in the DNA-binding domain of BetR. Having reconstructed this mutant in a clean background, we observed that this single amino acid change results in nearly complete loss of T6SS activity (Figure 4C). These data show that BetR controls T6SS-mediated bacterial antagonism across *S. marcescens* lineages 13, 12, and 9. By inferring the most recent common ancestor of these isolates (Figure 4A) and taking into account the conservation of the *betR-reg1-reg2* locus across *Serratia* (Figures 3 and S4), we predict that BetR regulation controls T6SS activity across at least *S. marcescens* lineages 9, 12, 14, 13, and 15, and most likely the entire species.

### The BetR system controls a phenotypic switch in *Serratia marcescens*

**Figure 5:**
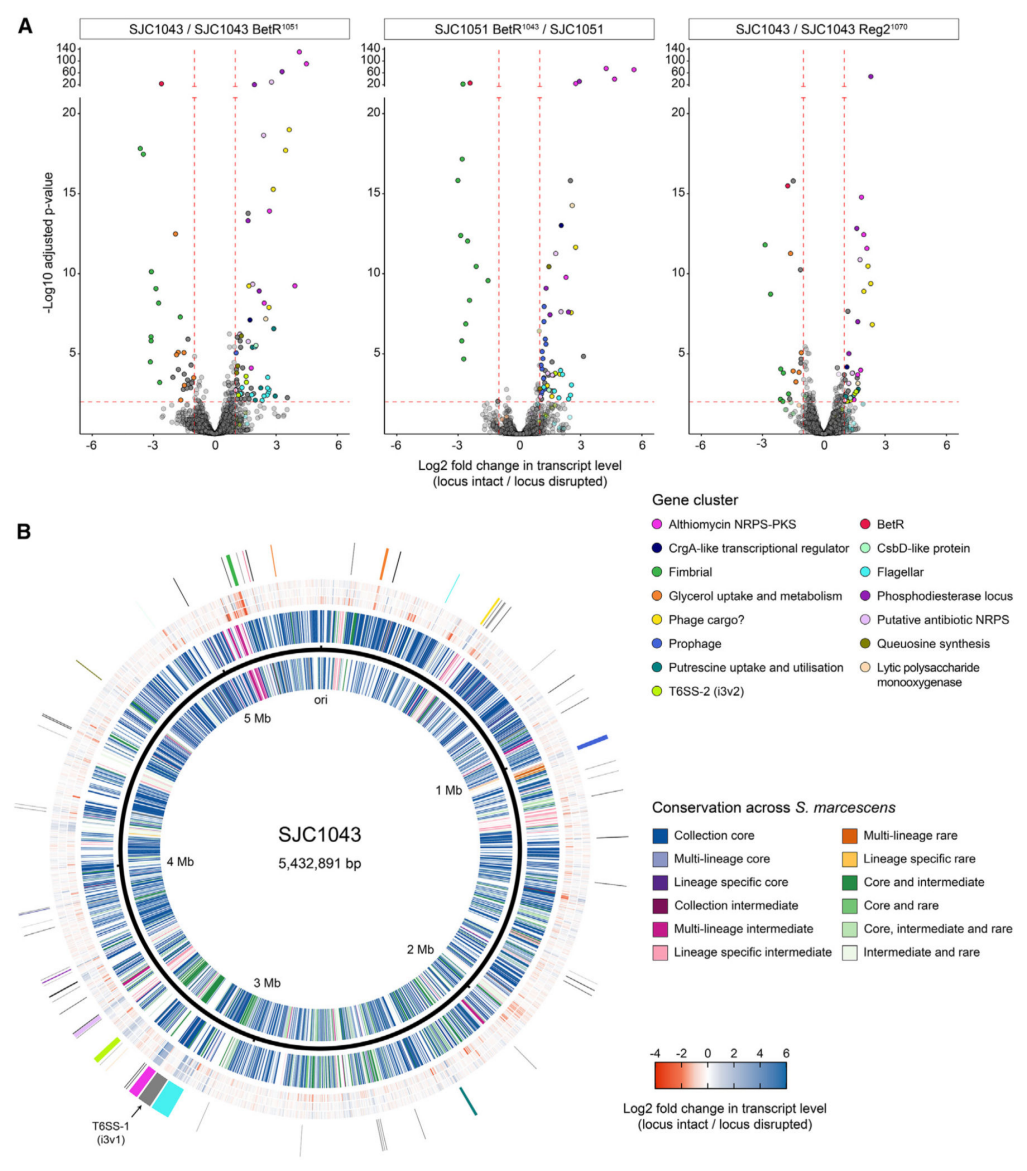
Transcriptomic analysis reveals that the BetR system regulates a defined set of core and lineage-specific genes, suggesting control of a lifestyle switch in *Serratia marcescens* (A) Volcano plots of RNA-seq-based comparisons between pairs of wild-type and otherwise-isogenic *betR* or *reg2* exchanged strains. In each case, the log_2_ fold change in transcript abundance was calculated as (*betR* locus intact/*betR* locus disrupted). Genes with significantly altered transcript levels in each comparison were defined as those with log_2_ fold change >1 or <−1 and adjusted *p* < 0.01 (*n* = 4 biological replicates), indicated by red dashed lines and solid color points. Genes within gene clusters of interest are colored according to the key, with other genes in dark gray. (B) BetR system-regulated genes shown in the context of their position on the SJC1043 chromosome and level of conservation across *S. marcescens*. Rings in the circular plot, from inside to out, show: (1) reverse-strand genes colored by conservation across *S. marcescens* using Twilight,^18^ according to the key; (2) black line representing the SJC1043 chromosome; (3) forward-strand genes colored by conservation (as ring 1); (4) log_2_ fold change in transcript level for comparison between SJC1043 and SJC1043 BetR^1051^ colored as a heatmap according to the key; (5) heatmap comparison of SJC1051 and SJC1051 BetR^1043^ (as ring 4); (6) heatmap comparison of SJC1043 and SJC1043 Reg2^1070^ (as ring 4); and (7) location of genes significantly altered in any of the three comparisons, colored as part A except that those without specific categories are in black. The position of the T6SS_i3v1 gene cluster (not transcriptionally altered) is shown in gray and labeled. See also Table S2 and Data S2.

Since BetR is predicted to act as a transcriptional regulator, we used RNA sequencing (RNA-seq) to compare the transcriptomes of strains with intact BetR systems (T6SS-active) against those with disrupted BetR systems (T6SS-inactive) to gain insight into the mode of T6SS regulation and other traits that might be co-regulated with the T6SS. Three two-way comparisons between otherwise isogenic strains were performed: wild-type SJC1043 vs. SJC1043 with the disrupted *betR* allele from SJC1051; wild-type SJC1043 vs. SJC1043 with disrupted *reg2* from SJC1070; and SJC1051 with intact *betR* from SJC1043 vs. wild-type SJC1051 (Figure 5A). The three comparisons were in strong accordance with each other. They revealed that transcripts significantly increased when the BetR system was intact included those from gene clusters encoding flagella, a cyclic AMP (cAMP) phosphodiesterase (CpdA1), althiomycin biosynthesis, another putative antibiotic NRPS, and the second T6SS, T6SS_i3v2, as well as those from certain putative regulatory, phage-associated, and metabolic genes. Conversely, when the BetR system was disrupted, transcription of a fimbrial gene cluster was strikingly increased, and genes for glycerol utilization and *betR* itself were also upregulated (Figure 5A; Table S2; Data S2). The BetR-regulated genes are distributed around the entire chromosome and do not cluster in a particular region of the genome (Figure 5B). However, there was no difference in transcript levels of genes encoding the primary T6SS, T6SS_i3v1, consistent with the data suggesting that activity of this T6SS is regulated post-translationally (Figure 2B).

Having observed BetR control of T6SS activity across multiple *S. marcescens* lineages, we examined the conservation of genes transcriptionally regulated by *betR*. Many of these gene groups are core to *S. marcescens*, including flagellar, fimbrial, glycerol utilization, queuosine synthesis, and putrescine utilization genes (Figure 5B; Table S2). However, other regulated genes or gene clusters are not core, including the althiomycin gene cluster, additional putative NRPSs, T6SS_i3v2, and *cpdA1*, which we previously identified as being encoded within a hypervariable region.^13^ Overall, the transcriptomic data suggest that the BetR-Reg1-Reg2 system positively regulates active competition and pioneering phenotypes while simultaneously negatively regulating adhesive and resilient phenotypes, representing a phenotypic switch that integrates both core and lineage-specific traits.

### Adhesion and antibiotic production phenotypes are regulated by BetR, while T6SS activity is influenced by cAMP levels but not fimbrial overexpression

**Figure 6:**
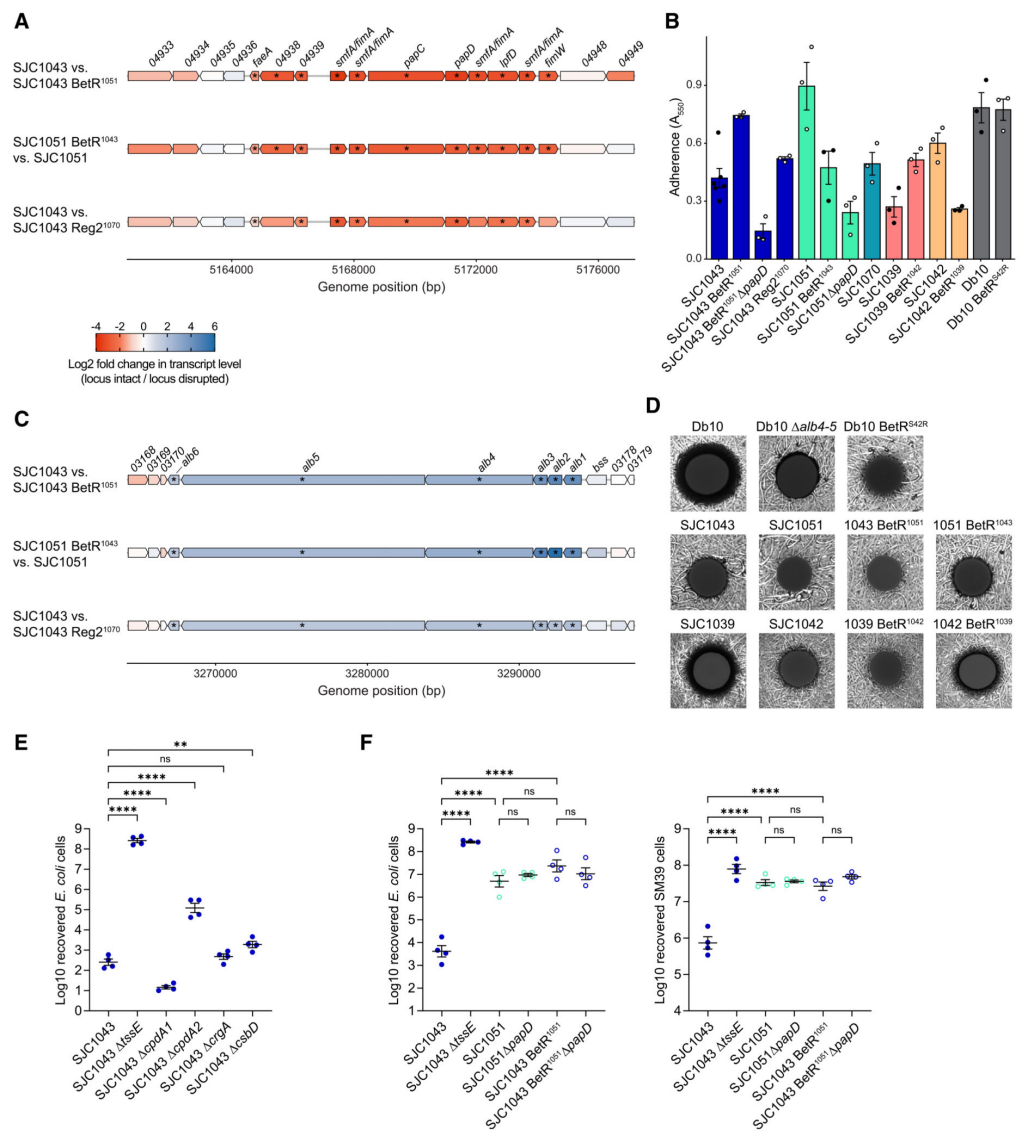
Phenotypic consequences of *betR* transcriptional regulation on fimbrial attachment, production of diffusible antimicrobials, and T6SS activation (A and C) Diagrams of the *betR*-regulated fimbrial gene cluster (A) and althiomycin gene cluster (C) in *S. marcescens* SJC1043, colored by the log_2_ fold change in transcript level observed in each comparison between strains with intact or disrupted *betR* locus. Genes with adjusted *p* < 0.01 in that comparison are shown by an asterisk. Numbers above genes indicate gene identifiers SJC1043_xxxxx. (B) Adhesion of the indicated strains of *S. marcescens* to an abiotic plastic surface, quantified using crystal violet staining (bars show mean ± SEM with individual data points overlaid). (D) Fluorescence images showing antibiosis haloes (zones of inhibition) when the indicated strains of *S. marcescens* were spotted on a lawn of *Bacillus subtilis* expressing GFP. (E and F) Recovery of *E. coli* or *S. marcescens* SM39 when co-cultured with wild-type *S. marcescens* SJC1043 or SJC1051, reciprocal *betR* allele exchanges in these backgrounds, or derivatives carrying in-frame deletions of *tssE, cpdA1, cpdA2, crgA, cbsD*, or *papD*, as indicated. Data are presented as mean ± SEM with individual data points overlaid (*n* = 4 biological replicates; ****p < 0.0001, **p < 0.01, ns not significant; one-way ANOVA with [E] Dunnett’s or [F] Tukey’s test; for clarity, only selected comparisons are displayed). Filled circles indicate isolates with *betR* and *reg2* intact, and open circles indicate isolates with *betR* or *reg2* disrupted. See also Figure S5.

We predicted that the markedly increased expression of fimbrial genes in *betR*-disrupted isolates (Figure 6A), alongside decreased expression of flagellar genes, would result in greater adherence, including to abiotic surfaces. Measuring abiotic surface adhesion experimentally confirmed that disrupted *betR* or *reg2* resulted in greater adherence, dependent on the Pap-like fimbriae whose transcription was increased in the RNA-seq (Figure 6B). BetR-dependent repression of adhesion was also observed in clinical isolates SJC1039 and SJC1042, but not in Db10, perhaps reflecting variation in BetR-dependent phenotypes across *S. marcescens* or compensation by another fimbrial gene cluster in the non-clinical lineage. Flagellar-dependent motility was also observed to be BetR-dependent in several lineages of *S. marcescens*, although SJC1043 did not display motility in the media tested (Figures S5A and S5B). Another key finding was that the althiomycin NRPS-PKS gene cluster and another putative antibiotic-producing NRPS are strongly positively regulated by the BetR system (Figures 5 and 6C; Table S2). Consistently, antibiosis haloes are produced on a lawn of *Bacillus subtilis* by *betR*-intact strains but not the corresponding *betR*-disrupted strains (Figure 6D). In Db10, the almost-complete loss of the antibiosis halo in an althiomycin mutant suggests that althiomycin represents the primary contribution to this phenotype. However, the althiomycin genes are not conserved in all *S. marcescens* (Figure 5B) and are not present in lineage 9, which includes SJC1039 and SJC1042. Nevertheless, a clear BetR-dependent zone of antibiosis is observed in these isolates (Figure 6D), implying that other gene clusters directing the production of antibiotic molecules can be brought under the control of the BetR system in *S. marcescens*.

Our initial observation was of BetR-dependent variation in T6SS activity. However, BetR does not alter transcription of the T6SS_i3v1 gene cluster or production of Hcp (Figures 2 and 5), implying that T6SS activity is influenced post-translationally, as a downstream consequence of the primary transcriptional impact of BetR. We identified several candidates for BetR-regulated genes whose products might affect T6SS activity, focusing on those conserved across lineages in which BetR was shown to control T6SS activity, and determined the impact of their deletion on T6SS-dependent antibacterial activity. Loss of the positively BetR-regulated CrgA-like transcriptional regulator or CbsD-like stress response protein resulted in no change or a small decrease, respectively, in activity (Figure 6E). One of the genes most strongly positively regulated by BetR, *cpdA1*, encodes a predicted cAMP phosphodiesterase, while *cpdA2*, encoding the second cAMP phosphodiesterase in SJC1043, is modestly negatively regulated (Table S2), suggesting that altered cAMP levels may contribute to BetR control of T6SS activity. Deletion of *cpdA1* led to a small increase in antibacterial activity, while deletion of *cpdA2*, or both together, led to a substantial decrease in T6SS activity (Figures 6E and S5C). These data suggest that the appropriate level of cAMP is required for full T6SS activity and that BetR regulation of *cpdA* genes may contribute to its impact on T6SS activity. However, regulation may depend on finely balanced cAMP levels or feedback control, since the T6SS phenotypes of the deletion mutants (with complete loss of CpdA) are in the opposite direction from what might be predicted based on how BetR modulates expression of the two *cpdA* genes. Finally, we determined whether the increased fimbrial expression observed in *betR*-disrupted isolates was responsible for inhibiting T6SS-dependent antibacterial activity, perhaps by increasing self-aggregation and spatial separation of attacker and target as observed recently.^20^ However, deletion of the essential fimbrial component *papD* did not cause any restoration of T6SS activity against *E. coli* or another strain of *S. marcescens* (Figure 6F). Overall, we conclude that BetR regulation of T6SS activity in *S. marcescens* is likely multifactorial but does not result from its repression of fimbrial expression.

### Loss of BetR leads to increased virulence in an *in vivo* infection model

**Figure 7:**
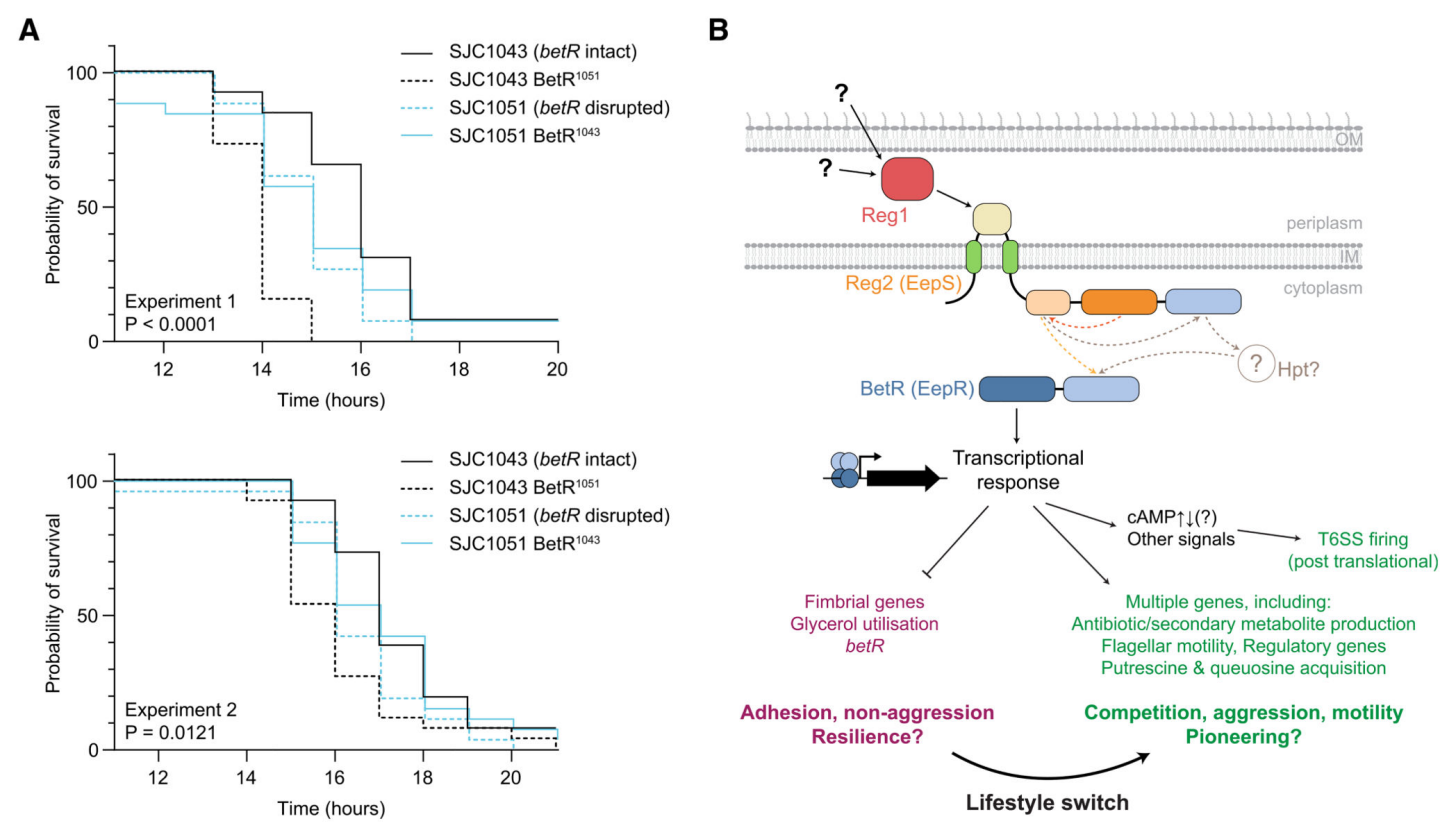
The BetR-dependent lifestyle switch affects host survival in an *in vivo* infection model (A) Two independent experiments showing the survival of *Galleria mellonella* following inoculation with 10 colony-forming units (CFU) of *S. marcescens* clinical isolates SJC1043, SJC1051, or their derivatives with exchanged *betR* alleles, and incubation at 37°C (*n* = 26 larvae for each strain; survival curves are significantly different by log-rank (Mantel-Cox) test with *p* values as shown). SJC1043 BetR^1051^ indicates SJC1043 with the *betR* allele from SJC1051 and vice versa. (B) Model of the BetR-mediated lifestyle switch in *Serratia*. In response to an unknown periplasmic signal, likely extracellular in origin, Reg1 interacts with the periplasmic sensing domain of Reg2, causing autophosphorylation and dimerization of the histidine kinase domains (phosphotransfer indicated by dashed arrows). This causes phosphorylation of the receiver domain of BetR, either directly from the Reg2 phosphoacceptor domain or via the Reg2 receiver domain and an unknown histidine-containing phosphotransfer protein (Hpt), leading to BetR dimerization and interaction of its DNA-binding domain with DNA, altering transcription of many target genes. The transcriptional response causes a concerted lifestyle switch from an adhesive, passive, and potentially resilient mode to an aggressive, motile, and likely pioneering mode via transcriptional regulation of multiple operons and post-translational regulation of T6SS_i3v1 activity. Protein domains are colored as in Figure 2; IM, inner membrane; OM, outer membrane.

Finally, to gain insight into whether disruption of *betR*, as observed in clinical isolates, has a direct impact on host infection by *S. marcescens*, we compared the virulence of wild-type SJC1043 with its derivative carrying the disrupted *betR* allele from SJC1051 (SJC1043 BetR^1051^) using the *Galleria mellonella* (waxmoth larvae) model.^21^ This revealed a significant and reproducible decrease in the survival of *Galleria* inoculated with the *betR*-disrupted strain compared with the wild type (*p* < 0.01 in two independent experiments, log-rank test), implying that loss of BetR can increase virulence in a host organism (Figure 7A). Restoration of the intact *betR* allele from SJC1043 into SJC1051 had a more modest impact on *Galleria* survival, but we observed a consistent trend of SJC1051 killing slightly faster than its BetR-intact derivative (Figure 7A). Taken together, the data indicate that the *betR*-controlled lifestyle switch can directly influence disease-causing interactions of *S. marcescens* with eukaryotic hosts.

## Discussion

In this study, integration of genomics with molecular phenotypic characterization has revealed that a genus-core two-component system, Reg1-Reg2-BetR, positively regulates competitive and motile phenotypes while negatively regulating sessile and potentially “hiding” behaviors in *S. marcescens*, apparently coordinating a lifestyle switch from “pioneering” to “resilient” (Figure 7B). Strikingly, we observed that loss-of-function mutations are highly enriched in *reg2* and *betR*, occur in different genetic lineages, and are only seen in isolates from clinical environments, suggesting that there is a clinically associated pressure selecting for one or more phenotypes resulting from non-functional Reg2-BetR.

It is currently unknown whether the selection of *betR* and *reg2* mutants occurs within patients, either during carriage or disease, or in the clinical abiotic environment. Nevertheless, fitness in all these contexts should contribute to the spread and/or pathogenicity of antimicrobial-resistant opportunistic bacteria. Our analysis, which showed that *betR* presented the most unique loss-of-function mutations observed exclusively in clinically associated *S. marcescens*, additionally highlighted that *sdeS*, encoding a negative regulator of a multidrug efflux pump, is also subject to frequent loss-of-function mutations in clinically derived *S. marcescens*. Mutations in genes equivalent to *sdeS* have been detected in *Pseudomonas aeruginosa* within the airways of cystic fibrosis patients,^22^ where the selection pressure is likely exposure to antibiotics. In the case of the BetR system, the precise nature of the clinical selection pressure(s) is less easily predicted, in part because our analysis is limited by available metadata and cannot distinguish between carriage, disease, or abiotic clinical isolates.

On one hand, the phenotypes manifest when BetR is inactivated, particularly increased adhesion due to fimbrial upregulation and flagellar downregulation, may result in greater biofilm formation^23^ and increased resistance to antimicrobials, such as antibiotics^24^ or detergents,^25,26^ generating selection for BetR inactivation in the patient or hospital environment. Alternatively, phenotypes positively regulated by intact BetR, such as actively firing T6SSs or increased flagellar expression, may increase fitness before or during initial infection but could be disadvantageous once established in the host, for example, due to a high degree of immunogenicity of flagellar or secreted T6SS proteins.^27–29^ The observation that inactivation of *betR* can lead to increased virulence in an *in vivo* infection model indicates that within-patient factors likely contribute to the selective advantage, for example, increased attachment to host cells, reduced immune system activation, or increased resistance to antimicrobial defenses. Perhaps most likely is a scenario whereby the selection favoring BetR inactivation is for a combination of individual phenotypic changes, explaining why the switch itself, rather than individual traits, is subject to such frequent mutation in the clinic. Furthermore, we believe that the selective pressure for *betR* inactivation in a clinical environment is unlikely to be solely due to factors that cause increased virulence in the *Galleria* model. It may also involve factors important for environmental persistence or interaction with the adaptive immune system, which promote survival and transmission. Since two-component regulators often control multifaceted responses involved in clinical adaptation, clinically selected mutations in such systems are frequently reported, for example, phase variation in *Bordetella pertussis*^30^ and antibiotic resistance resulting from mutations in *pmrAB* in *Klebsiella pneumoniae* or *phoPQ* in *P. aeruginosa*.^31,32^

The BetR-dependent regulon appears to vary across *S. marcescens*, suggesting a modular system whereby variable non-core traits, such as different antibiotic biosynthesis genes, can be regulated coordinately with core genes to promote the overarching lifestyle switch. This is supported by the observation of BetR-dependent regulation of antibiotic production in strains with variable NRPSs and by the fact that a number of the genes most affected by BetR are not conserved across the species. Such flexibility may facilitate the ready acquisition and exchange of genes promoting inter-bacterial competition, while the conservation of *reg1-reg2-betR* across the genus implies that the BetR “pan-regulon” may be large. Our RNA-seq data indicate that BetR regulates its own expression in a negative feedback loop; however, the signal activating the system remains to be identified. We predict that the periplasmic protein Reg1 will interact with the periplasmic domain of Reg2 to activate or repress its kinase function (Figure 7B). This interaction is likely to transduce an extracellular signal, either via binding of a periplasmic small molecule or a condition-sensitive conformational change in Reg1.

BetR-Reg2 were previously identified as regulators (called EepR-EepS) promoting the production of secondary metabolites (the antimicrobial pigment prodigiosin and biosurfactant serrawettin) and several secreted enzymes in other isolates of *S. marcescens*.^33,34^ While none of the strains studied here produce prodigiosin, which is limited to lineage 15,^13^ the earlier findings are consistent with our observation of BetR regulating multiple secondary metabolites and promoting motility. More broadly, they support the idea that the BetR system regulates variable sets of genes across the species. EepR-EepS (BetR-Reg2) were also shown to be negatively regulated by cAMP-bound cAMP-receptor protein (cAMP-CRP).^34^ Separately, cAMP-CRP was shown to upregulate flagellar gene transcription and repress fimbrial-mediated biofilm formation in *S. marcescens* PIC3611.^35,36^ Here, in SJC1043, we found that BetR oppositely regulates the expression of two cAMP phosphodiesterases, whose activity will counteract the production of cAMP by adenylate cyclase which occurs in the absence of catabolite repression. Taken together, this suggests that the BetR system and cAMP signaling are integrated in a finely tuned network where differential activation of the BetR system or changes in carbon source availability can alter cAMP levels and cAMP-CRP-dependent gene expression in response to environmental changes. Nevertheless, we believe that this is only one aspect of the complex regulation exerted by BetR, given that BetR has been shown to regulate several genes directly^33,34^ and that the strongly BetR-regulated phosphodiesterase, CpdA1, is not conserved across the species.

An interesting question is why there is such strong clinical selection pressure for BetR mutations that appear to terminally inactivate a central lifestyle switch and remove the ability of *S. marcescens* to respond appropriately to changes in conditions. One possible explanation is that *betR* mutation and selection represent an immune evasion strategy, particularly if several BetR-regulated extracellular proteins (e.g., Hcp, exoenzymes, and/or flagella) are immunogenic. Our *in vivo* data indicate that *betR* inactivation can provide a selective benefit in the host during infection and would be consistent with the *betR* mutant activating the *Galleria* innate immune system less effectively. In this model, upon exit from the host, the *betR*-inactive mutants are less fit in an environmental context and are outcompeted. Consistent with this idea, we have not observed any descendants from *betR*/*reg2* mutants in available genome sequences. Therefore, these may be “dead-end” mutants, incompatible with the lifestyle of an opportunistic pathogen, unless the mutations are somehow reversible, which could result in a process similar to phase variation. Alternatively, *betR/reg2* mutants may arise as part of a mixed community of *S. marcescens*, whereby these mutants promote biofilm formation while the remainder of the population maintain intact BetR systems and protect the community through antibiotic production and T6SS activity. Sequenced clinical isolates may represent single cultured cells from a potentially genetically heterogeneous population. Therefore, it is possible that populations of *S. marcescens* in clinical settings may include *betR/reg2* mutants co-existing with *betR/reg2*-intact clones and that the phenotype(s) of the mutants are selected by their contribution to the common good or in the context of being “cheaters” protected by others. Furthermore, a recent study reported very low expression of *eepR/betR* in a clinical isolate of *S. marcescens*, implying that *betR* signaling may also be turned off by loss of *betR* expression rather than BetR/Reg2 function in some clinical isolates.^37^

Interestingly, repression of T6SS activity, which is one of the major consequences of *betR/reg2* mutation, promotes spread of multidrug resistance plasmids in *Acinetobacter*^38^ and thus may be another factor contributing to acquisition of these mutations. T6SS inactivation has also been observed in isolates of *Bacteriodes fragilis* in the human gut microbiota^39^ and during chronic *P. aeruginosa* infection in cystic fibrosis patients.^40^ Nevertheless, similar to what we observe in *S. marcescens*, irreversible loss of the T6SS has not become fixed in major lineages of these organisms, implying there is a long-term benefit to retention. Our data show that BetR is a key, conserved regulator of T6SSs in *S. marcescens*, and, in the case of the core i3v1 system, this control operates via a post-translational mechanism that likely involves altered cAMP but requires future elucidation. Moreover, this regulation coordinates activity of the T6SS, effective against gram-negative and fungal competitors, with expression of althiomycin and other antibiotics effective against gram-positive bacteria, making *S. marcescens* a formidable competitor.

In conclusion, this work has revealed that competitive and pioneering phenotypes, including T6SS and antibiotic production, are co-regulated in *S. marcescens* by a highly conserved two-component regulatory system that controls a modular lifestyle switch and whose inactivation is strongly selected in clinically derived isolates. T6SS_i3v1 is highly conserved across multiple *Serratia* species, implying that while there may be short-term benefit in its inactivation in certain niches, there is an evolutionary advantage in its long-term retention for members of the genus that circulate across a diverse range of niches. Furthermore, this study suggests that examination of whole genome data for the genes most subject to independent severe mutations in clinically derived isolates should reveal similar critical genes for adaptation in other opportunistic pathogens.

## Resource Availability

### Lead contact

Requests for information and resources should be directed to the lead contact, Sarah Coulthurst (s.j.coulthurst@dundee.ac.uk).

### Materials availability

Bacterial strains and plasmids are available from the lead contact.

## Star★Methods

Detailed methods are provided in the online version of this paper and include the following:

- KEY RESOURCES TABLE
- EXPERIMENTAL MODEL AND STUDY PARTICIPANT DETAILS
  ○ *Galleria mellonella* larvae
  ○ Bacterial strains and plasmids
- METHOD DETAILS
  ○ Co-culture assays for T6SS-mediated antibacterial activity
  ○ Immunoblot assay for Hcp1 production and secretion
  ○ RNA sequencing
  ○ RNAseq analysis
  ○ Adhesion assay
  ○ Antibiosis assay
  ○ Motility assay
  ○ Galleria mellonella virulence assay
  ○ T6SS searching and filtering
  ○ Variant calling and chromosomal synteny analysis
  ○ Phylogenetic tree construction
  ○ Plasmid identification
  ○ Protein domain and DNA sequence analysis
  ○ Data visualisation
- QUANTIFICATION AND STATISTICAL ANALYSIS

## Star★Methods

### Key Resources Table

**Table:** 

REAGENT or RESOURCE	SOURCE	IDENTIFIER
Antibodies		
Polyclonal rabbit anti-Hcp primary	Murdoch et al.^10^	N/A
HRP-conjugated goat anti-rabbit secondary	BioRad Laboratories	#0170-6515
Bacterial and virus strains		
For strains used in this study see Table S3	see Table S3	see Table S3
Chemicals, peptides, and recombinant proteins		
TRIzol™ LS Reagent	Invitrogen (ThermoFisher Scientific)	#10296010
Select agar	Invitrogen (ThermoFisher Scientific)	#30391023
Bacto agar	BD Biosciences	#214010
Bacto tryptone	ThermoFisher Scientific	#211705
Yeast extract	Merck-Millipore	#1037530
Critical commercial assays		
Enhanced chemiluminescent detection kit	Millipore	#11546345
Deposited data		
Raw RNAseq data	This study	GEO: GSE273522 (https://www.ncbi.nlm.nih.gov/geo/query/acc.cgi?acc=GSE273522)
*Serratia* genome sequences and pangenome	Williamset al.^13^,^41^	https://pubmed.ncbi.nlm.nih.gov/36057639/ https://doi.org/10.6084/m9.figshare.18051824.v2
Experimental models: Organisms/strains		
*Galleria mellonella*	LiveFood UK Ltd	N/A
Oligonucleotides		
For primer sequences see Table S4	see Table S4	see Table S4
Recombinant DNA		
For Plasmids used in this study see Table S3	see Table S3	see Table S3
Software and algorithms		
Multi-bacpipe V0.8.0	Predeus	https://github.com/apredeus/multi-bacpipe
STAR v2.6.0c	Dobin et al.^42^	https://github.com/alexdobin/STAR
featureCounts v1.6.4	Liao et al.^43^	https://subread.sourceforge.net
Phantasus	Kleverov et al.^44^	https://artyomovlab.wustl.edu/phantasus
DESeq2 V1.30.1	Love et al.^45^	https://bioconductor.org/packages/release/bioc/html/DESeq2.html
LAS software V2.7.1	Leica Microsystems	https://www.leica-microsystems.com/products/microscope-software/p/leica-las-x-ls
Hamburger V0.2.0	Williams^46^	https://www.github.com/djw533/hamburger
BLAST V2.2.31 +	NIH^47^	https://blast.ncbi.nlm.nih.gov/doc/blast-help/downloadblastdata.html
GenoplotR V0.8.1188	Guy et al.^48^	https://genoplotr.r-forge.r-project.org
Canu v1.8	Koren et al.^49^	https://github.com/marbl/canu
snippy V4.3.3	Seemann	https://github.com/tseemann/snippy
snpEff v4.3	Cingolani et al.^50^	https://pcingola.github.io/SnpEff
SNP-sites V2.4.1	Page et al.^51^	https://github.com/sanger-pathogens/snp-sites
Phaster	Arndt et al.^52^	https://phaster.ca
IQtree v.1.6.10	Nyugen et al.^53^	https://github.com/iqtree
Mash V2.1.1	Ondov et al.^54^	https://github.com/marbl/Mash
fastANI_to_clusters.py	Williams	https://github.com/djw533/Serratia_genus_paper/analysis_scripts
InterProScan	EMBL-EBI^55^	https://www.ebi.ac.uk/interpro/search/sequence
ggtree V2.4.290	Yu et al.^56^	https://bioconductor.org/packages/release/bioc/html/ggtree.html
gggenes V0.4.1	Wilkins^57^	https://cran.r-project.org/web/packages/gggenes/vignettes/introduction-to-gggenes.html
ggplot2 V3.3.583	Wickham^58^	https://ggplot2.tidyverse.org
Cytoscape V3.7.178	Shannon et al.^59^	https://cytoscape.org
fasttree V2.1.8	Price et al.^60^	http://www.microbesonline.org/fasttree/
imageJ v1.50e	Schneider et al.^61^	https://imagej.net/downloads
GraphPad Prism V9.5.1	GraphPad	https://www.graphpad.com/
R V4.0.3		https://cran.r-project.org/

### Experimental Model And Study Participant Details

#### *Galleria mellonella* larvae

*G. mellonella* last-instar larvae were purchased (LiveFood UK Ltd) and stored in wood chips at room temperature before and throughout the experimental setup. Larvae were sorted prior to experiments and any larvae already showing signs of melanisation or larvae of extreme size were excluded.

#### Bacterial strains and plasmids

Bacterial strains and plasmids used in this study are detailed in Table S3. We note that *S. marcescens* Db10 is used for all experimental work, whilst *S. marcescens* Db11, immediately derived from Db10, is the strain whose whole genome sequence was determined.^14^ Since the two strains differ by only a single base pair mutation in *rpsL*,^14^ the genome sequence for Db11 can be used interchangeably for Db10. Strains of *S. marcescens* carrying precise chromosomal mutations, namely in-frame deletions and reconstructions of wild-type or disrupted alleles of regulatory genes, were generated by allelic exchange using the suicide plasmid pKNG101.^10,62^ Primer sequences and details of plasmid construction are provided in Table S4. Strains of *S. marcescens* were grown at 30°C or 37°C on LB agar (LBA, 10 g/L tryptone, 5 g/L yeast extract, 10 g/L NaCl, 18 g/L Select agar) in liquid LB (10 g/L tryptone, 5 g/L yeast extract, 5 g/L NaCl), whilst for *Escherichia coli* growth was at 37°C and for *Pseudomonas fluorescens* it was at 30°C, also in LB or on LBA. Minimal media contained 40 mM K_2_HPO_4_, 15 mM KH_2_PO_4_, 0.1% (NH_4_(_2_SO_4_, 0.4 mM MgSO_4_, 0.2% glucose. When required, media were supplemented with kanamycin (Kn) 100 μg/ml or streptomycin (Sm) 100 μg/ml.

### Method Details

#### Co-culture assays for T6SS-mediated antibacterial activity

Using our standard co-culture assay,^63^ cell suspensions of ‘attacker’ strains of *S. marcescens* and the required target strain were normalised to OD_600_ 0.5 in LB, mixed at a 1:1 ratio, and 25 μL of the mixture was spotted onto solid LBA and then grown for 4 h at 37°C (*E. coli* target) or 30°C (*P. fluorescens* target). Co-cultures of clinical isolates with *S. marcescens* SM39 were at 30°C, except for Figure 6F when 37°C was used. Following the co-culture, cells were recovered into 1 ml LB and the number of surviving target cells was enumerated by serial dilution and viable counts on Kn-supplemented LB agar (*E. coli* target) or Sm-supplemented LB agar (*P. fluorescens* and SM39 targets)

#### Immunoblot assay for Hcp1 production and secretion

Anti-Hcp immunoblotting was used to detect Hcp1 in cellular and secreted (supernatant) fractions. 25 ml cultures were grown for 5 h in LB at 30°C or 37°C. To prepare cellular samples, cells from 100 μL culture were isolated by centrifugation (20 min, 15,000*g*), resuspended in 200 μL of 2x SDS sample buffer (100 mM Tris-HCl pH 6.8, 3.2% SDS, 3.2 mM EDTA, 16% glycerol, 0.2 mg/mL bromophenol blue, 2.5% β-mercaptoethanol) and boiled for 10 min. To prepare secreted protein samples, cell-free supernatant was recovered by centrifugation of 1 ml culture (10 min, 15,000*g*) and 100 μL supernatant was mixed with 100 μL 2x SDS sample buffer. The cellular and secreted protein samples, normalised between strains and replicates for OD_600_ of the culture, were separated by 15% SDS-PAGE and electroblotted onto polyvinylidine fluoride (PVDF, Millipore). Hcp1 was detected by hybridisation of the primary antibody, polyclonal rabbit anti-Hcp^64^ (1:6000), followed by the secondary antibody, HRP-conjugated goat anti-rabbit (BioRad #0170-6515; 1:10000), and then the use of an enhanced chemiluminescent detection kit (Millipore).

#### RNA sequencing

For isolation of total RNA, four independent biological replicates of each relevant strain of *S. marcescens* were grown in 25 ml LB at 37°C, 200 rpm to an OD_600_ of 2.5. Cultures were mixed with 10 ml ice-cold stop solution (19% ethanol, 1% acidic phenol pH 4.3) and incubated on ice for 30 min. Cells were then recovered by centrifugation, resuspended in 1 mL TRIzol and transferred into a 2 mL phase lock tube (QuantaBio #2302830), and 400 μL chloroform was added and immediately mixed for 10 s. After incubation for 2 min at room temperature, the suspension was separated by centrifugation for 15 min at 20,000 *g*. The upper phase containing the RNA (∼500 μL) was transferred into a 1.5 mL microcentrifuge tube, 450 μL isopropanol was added, and the suspension was mixed by inverting and then incubated at room temperature for 30 min. The precipitated RNA was recovered by centrifugation for 30 min at 20,000 *g* and washed in 350 μL 70% ethanol. Following a further centrifugation step (10 min, 20,000 *g*), the pellet was air-dried, resuspended in RNase-free water and stored at -80°C. RNA samples were sequenced by Vertis Biotechnologie AG (Germany): following DNase treatment, strand-specific cDNA libraries for whole transcriptome sequencing (including sRNA > 70 nt) were prepared and sequenced by Illumina NextSeq 500).

#### RNAseq analysis

RNA-Seq data quality control, alignment, quantification, and statistics calculations were done using the “simple” workflow of Bacpipe RNA-seq processing pipeline v0.8.0 (https://github.com/apredeus/multi-bacpipe). The finished genome sequence (including three plasmids) and genome annotation of *S. marcescens* strain SJC1043 was sequenced, assembled and annotated previously^13^ and is available on NCBI Genbank (assembly ID GCF_946406265.1). tRNA and rRNA features were taken from this annotation. Basic read quality control was performed with FastQC v0.11.8. Twelve RNA-seq samples containing 14.81-19.88 millions of single-end 75 bp Illumina reads were aligned to the genome sequence using STAR v2.6.0c^42^ using “–alignIntronMin 20 –alignIntronMax 19 –outFilterMultimapNmax 20” options. For RNA-seq samples, 98.6-99.5% reads were mapped successfully; 48.2-74.7% of reads mapped to rRNA operons, tRNA operons or more than one location and were filtered off. The remaining 24.3-50.8% of initial reads mapped uniquely to the rest of the genome, resulting in 4.06-9.07M reads uniquely aligned to the genome for each sample.

For RNA-seq quantification, a processed GFF file was generated by Bacpipe, where all features of interest (rRNA and tRNA removed) were listed as a “gene”, with each gene identified by SJC1043 locus tag. The resulting GFF file contained 5086 features (4974 protein coding, 112 non-coding RNA). Following this, read counting was done by featureCounts v1.6.4,^43^ using options “-O -M –fraction -t gene -g ID -s 1”. Overall, 14.7-34.7% of initial reads were assigned to an annotated feature.

Variation in raw transcript counts per gene between replicates was assessed using the online web tool Phantasus.^44^ Raw transcript counts were log_2_ transformed and quantile normalised within Phantasus. Principal component analysis was used to assess variation between replicates. Based on this, replicate 3 for strain GM162 was determined to be an outlier and discarded from further analysis (Data S2).

Differential expression analysis was performed using DESeq2 v1.30.1^45^ with default settings. False discovery rate (FDR) adjusted p values were obtained using the Benjamini–Hochberg procedure within DESeq2. Strains SJC1043, SJC1051, GM161, and GM170 were present in 4 biological replicates, whilst GM162 was present in 3 biological replicates, as described above. A gene was considered to be differentially expressed if the absolute value of its log_2_ fold change was at least 1, and adjusted p-value was less than 0.01.

#### Adhesion assay

Overnight cultures of the required strains of *S. marcescens* strains grown at 30°C in minimal media were diluted to an OD_600_ of 0.02 in minimal media, 200 μL aliquots of each diluted culture were placed in eight wells of a 96-well microtitre plate (Greiner Bio-one #655 180), and the plate was incubated in a humid environment for 72 hr at 30 °C. To develop the assay, culture medium was removed from the wells by aspiration, the wells were washed with 250 μL deionised water and then the wells were filled with 240 μL 0.1 % (v/v) crystal violet and allowed to stain for 12 minutes. The crystal violet was removed by aspiration and the wells washed with 3x400 μL deionised water to remove non-attached material. Next, 250 μL 50 % ethanol was added to each well and incubated for 1 hr to allow the crystal violet staining adhered cells to dissolve. Finally the A_550_ for each well was measured in a Synergy 2 plate reader (Biotek) and the A_550_ value for each strain in that assay calculated as the mean of the eight wells (equivalent to pooling the wells).

#### Antibiosis assay

The indicator strain of *Bacillus subtilis* (NCIB3610 with P_hy-spac_-*gfpmut2*) was grown at 30°C in LB to early stationary phase and 100 μL of the culture diluted to an OD_600_ of 1 was spread onto an LBA plate. The strains of *S. marcescens* being tested for antibiotic production were grown to early stationary phase at 30°C in LB and 5 μL of culture diluted to an OD_600_ of 2 was spotted on the indicator lawn. The plates were incubated for approximately 16 hr at 30°C prior to imaging. Images were captured using a Leica MZ16 FA stereoscope (Leica) using LAS software version 2.7.1. For each strain, two independent repeats were carried out.

#### Motility assay

Swimming motility was measured in 25 ml motility agar (10 g/L tryptone, 5 g/L yeast extract, 5 g/L NaCl, 3g/L Bacto agar) in a 9 cm petri dish. Agar plates were dried for 30 minutes at room temperature in a laminar flow hood. Plates were inoculated with 2 μL of an overnight culture (grown at 37°C for 16 hours in LB) by injection deep into the semisolid agar. Plates were then incubated at 37°C for 7 or 20 hr and imaged on a VWR Chemi Premium imager using the ‘manual’ function. The area of the swim haloes was then calculated using ImageJ v1.50e, specifically by using the ‘measure’ function, relative to the size of the plate.

#### Galleria mellonella virulence assay

The invertebrate virulence model *Galleria mellonella* was used according to a protocol developed previously.^21^ In each experiment, each strain of *S. marcescens* to be tested was used to inoculate a group of 26 larvae of *G. mellonella*. Prior to injection, the larvae were sterilised by briefly rolling in 70% ethanol. Once approximately 90% of larvae had regained activity, each larva in the group was inoculated with a suspension of 10 CFU of *S. marcescens* in 10 μL phosphate-buffered saline (PBS), by injection into the rear right proleg using a sterilised Hamilton syringe. Two groups of control larvae were included, one injected with 10 μL sterile PBS prior to the bacterial inoculations to ensure minimal trauma was being caused by the injection procedure, and one injected with 10 μL sterile PBS after the bacterial inoculations to ensure no cross-contamination between groups. Following injection, larvae were incubated at 37°C. Survival was monitored hourly from 11 hours onwards. Larvae were considered to be dead when melanised and showing no signs of movement in response to external stimulus. Two independent experiments, performed on different occasions, are reported.

#### T6SS searching and filtering

Predicted T6SSs in annotated *Serratia* genomes were identified using Hamburger v0.2.0.^46^ Specifically, annotated genomes were scanned using hmmsearch from the HMMER3 suite to find loci where at least four core T6SS genes were present with no more than twelve non T6SS-core genes in between them. Identified T6SS loci were then subtyped by comparing them to a reference set of experimentally determined T6SSs obtained from the SecRET6 database,^65^ in addition to a manually curated set of other experimentally determined and predicted T6SSs (Figure S6; Table S5) Subtyping was performed by extracting TssB and TssC sequences from T6SSs identified by Hamburger and aligning them with TssB and TssC sequences from the reference set. Comparison of concatenated TssBC sequences between the reference and *Serratia* T6SSs through phylogenetic analysis using fasttree v2.1.8^60^ with default settings was used to assign T6SS subtypes to the identified T6SSs in *Serratia*, by taking all descendents from the most recent common ancestor of the reference TssBC sequences for each of the six proteobacterial T6SS subtypes (i1, i2, i3, i4a, i4b, i5) (Figure S7). Identified T6SSs that are situated outside of one of these defined proteobacterial T6SS subtypes were classed as undetermined. Further subdivision of subtype i3 into i3v1, i3v2 and i3v3 was based on phylogenetic position and manual comparison of gene composition and order within the T6SS loci. T6SS loci lacking intact *tssB* and/or *tssC* genes were classed as undetermined. Specific genomic positions of *Serratia* T6SS loci were recorded and annotated sequences of the individual T6SSs were extracted by Hamburger. The identified i3v1 T6SSs homologous to the model T6SS in *S. marcescens* Db10 were compared relative to the *Serratia* genus phylogeny and at the nucleotide sequence level. Representative copies of the i3v1 T6SS across *Serratia* were compared in a pairwise fashion by blastn from the BLAST 2.2.31+ suite. Comparisons were visualised using genoplotR.^48^

#### Variant calling and chromosomal synteny analysis

Reads associated with *S. marcescens* assemblies^13^ were downloaded from the ftp server for the ENA (http://ftp.sra.ebi.ac.uk/). Assemblies for which reads could not be located were shredded into simulated paired end reads using fastqSimulate from Canu v1.8.^49^ fastqSimulate was used with the following parameters: 75 bp simulated paired end reads; a shear size of 200 bp; 100x coverage; and a mismatch, error rate, and insertion likelihood of 0. Variants of *S. marcescens* genomes relative to the complete chromosomal sequence of SJC1043 were then discovered using snippy v4.3.3 (https://github.com/tseemann/snippy). Severity of the variants in relation to the predicted CDSs of SJC1043 were predicted using SnpEff v4.3.^50^ Two individual read sets with implausibly high numbers of high-severity mutations, when compared to the patristic distance to SJC1043 in the *S. marcescens* phylogeny, were considered outliers and were discarded from further analysis. A core SNP alignment was then created using snippy-core from snippy, with default settings. SNP-sites v2.4.1^51^ was then used to extract an alignment of polymorphic sites exclusively containing A, T, C, and G for tree drawing. Comparison between whole chromosomes was performed using blastn,^47^ using option “-task megablast”. Prophage regions were predicted by Phaster^52^ and from annotations and pangenome analysis performed previously.^13^

#### Phylogenetic tree construction

Core SNP alignments of the thirteen closely-related isolates were calculated and filtered using Snippy v4.3.3 and SNP-sites v.2.4.1 as above. The resulting alignment was 5,245,554 bp in length. IQtree v.1.6.10^53^ was then used for maximum-likelihood tree construction using 1000 ultrafast bootstraps^66^ using the TIM2e + ASC + R4 model chosen using modelfinder.^67^ Both the ultrafast bootstraps and modelfinder were implemented in IQtree.

#### Plasmid identification

Presence and absence of typed plasmids carried by the 13 genomes closely analysed in this study was determined using the 664 *Serratia* genomes dataset published previously.^13^ In order to find all possible plasmids, including both previously-typed plasmids and untyped extra-chromosomal circular contigs, all circularised contigs in genome assemblies for the 13 genomes analysed were compared to one another using Mash v2.1.1,^54^ and then clustered at a distance of 0.05 using the python script fastANI_to_clusters.py (https://github.com/djw533/Serratia_genus_paper/analysis_scripts).^13^ Clusters were then compared to the typed plasmids in the *Serratia* dataset. Untyped extra-chromosomal circular contigs were then compared to known plasmids using blastn.^47^

#### Protein domain and DNA sequence analysis

Protein sequences were analysed using the InterProScan webserver.^55^ Putative insertion sequences were identified through comparison using blastn from the blastn suite webservice.^47^

#### Data visualisation

Phylogenetic trees were visualised using the R package ggtree v2.4.290.^56^ Synteny of regions of bacterial genomes extracted by Hamburger and of whole chromosomes was visualised using the R package genoplotR v0.8.1188.^48^ Genetic organisation of genes were plotted using the R package gggenes v0.4.1.^57^ Other plots were created using the R package ggplot2 v3.3.583^58^ from the tidyverse package.^68^ Networks were viewed using Cytoscape v3.7.178.^59^

### Quantification And Statistical Analysis

All figure legends specify the experimental *n* and statistical analysis used. Data for all experiments displayed as bar graphs represent the mean and the standard error of the mean, with individual data points overlaid. For co-culture assays, significant differences in the number of recovered target cells between different attacking strains were determined using one-way ANOVA with Tukey’s or Dunnett’s post-tests, with four biological replicates. For analysis of motility assays, the area of the swim haloes was quantified using ImageJ v1.50e and comparisons between pairs of strains was performed using a students’ T-test, with three biological repeats. The above statistical analyses were performed using GraphPad Prism. For analysis of RNAseq data, which included four biological replicates for each strain, PCA analysis was performed on log_2_ transformed and quantile normalised data within phantasus^44^ and any outliers were removed from further analysis. Differential expression analysis was performed using DESeq2 v1.30.1^45^ with default settings. False discovery rate (FDR) adjusted p values were obtained using the Benjamini–Hochberg procedure within DESeq2. For the *Galleria mellonella* virulence assays, a group size of 26 was chosen based on the experimental group size calculation reported previously for the use of the same model and experimental protocol for *P. aeruginosa* PA01^21^ and pilot experiments indicating similar virulence in the model for the *S. marcescens* clinical isolates. The Log-rank (Mantel-Cox) test was used to test for significant differences in survival (GraphPad Prism).

## Supplementary Material

**Supplemental Information**

Supplemental information can be found online at https://doi.org/10.1016/j.chom.2025.01.001.

Supplementary data

Supplementary Data S1

Supplementary Data S2

## Data Availability

RNA-seq data have been deposited in the GEO repository with accession number GEO: GSE273522. This paper analyzes existing, publicly available genome and pangenome data, accessible at https://doi.org/10.6084/m9.figshare.18051824.v2. Other data reported in this paper are included within the main paper and supplemental material or available from the lead contact upon request. This paper does not report original code. Any additional information required to reanalyze the data reported in this paper is available from the lead contact upon request.

## In brief

Williams et al. discover that frequently occurring inactivating mutations in a conserved regulatory system lead to loss of competitive and pioneering behavior in clinical isolates of *Serratia marcescens*, including type VI secretion system activity, antibiotic production, and motility. This regulatory system controls a lifestyle switch subject to strong clinically associated selection pressure.

## Highlights

- One i3 T6SS is highly conserved across *Serratia*, while other T6SSs occur variably
- Mutations in a genus-core two-component regulatory system prevent T6SS activity
- This regulatory system is frequently inactivated, exclusively in clinical isolates
- The system controls a switch from adherent to competitive and motile behaviors
