# Supplementary material for: Competitive behaviors in Serratia marcescens are coordinately regulated by a lifestyle switch frequently inactivated in the clinical environment
Source: Cell Host Microbe. Author manuscript; Available in PMC 2026 Mar 14. (PMC7618870; doi:10.1016/j.chom.2025.01.001)
Supplement: Supplementary data [file EMS212892-supplement-Supplementary_data.pdf]

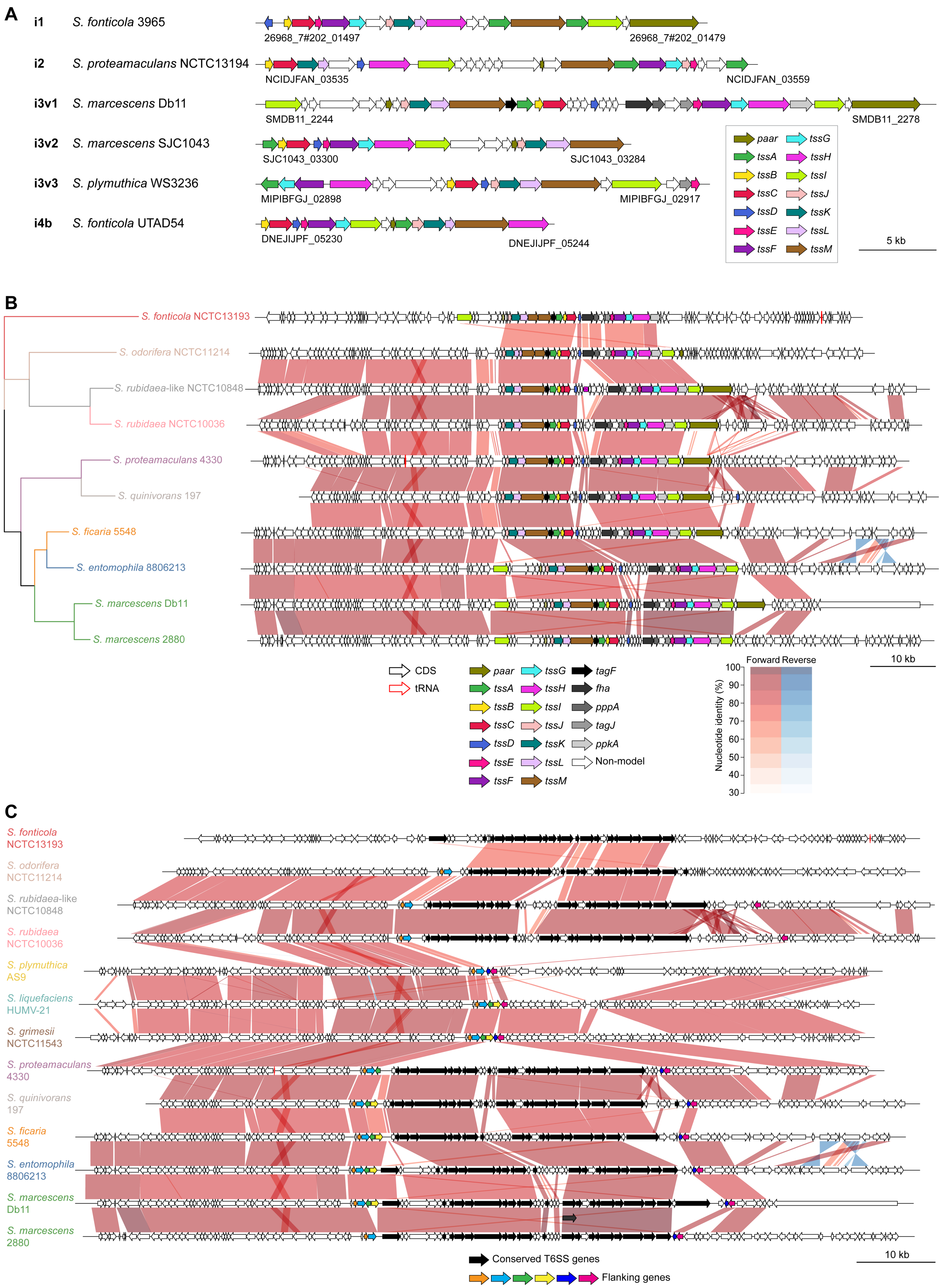

A
i1 S. fonticola 3965
26968_7#202_01497
26968_7#202_01479
i2 S. proteamaculans NCTC13194
NCIDJFAN_03535
NCIDJFAN_03559
i3v1 S. marcescens Db11
SMDB11_2244
SMDB11_2278
i3v2 S. marcescens SJC1043
SJC1043_03300
SJC1043_03284
i3v3 S. plymuthica WS3236
MIPIBFGJ_02898
MIPIBFGJ_02917
i4b S. fonticola UTAD54
DNEJIJPF_05230
DNEJIJPF_05244
paar tssG
tssA tssH
tssB tssI
tssC tssJ
tssD tssK
tssE tssL
tssF tssM
5 kb
B
S. fonticola NCTC13193
S. odorifera NCTC11214
S. rubidaea-like NCTC10848
S. rubidaea NCTC10036
S. proteamaculans 4330
S. quinivorans 197
S. ficaria 5548
S. entomophila 8806213
S. marcescens Db11
S. marcescens 2880
CDS
tRNA
paar tssG tagF
tssA tssH fha
tssB tssI pppA
tssC tssJ tagJ
tssD tssK ppkA
tssE tssL Non-model
tssF tssM
Forward Reverse
Nucleotide identity (%)
100
90
80
70
60
50
40
30
10 kb
C
S. fonticola NCTC13193
S. odorifera NCTC11214
S. rubidaea-like NCTC10848
S. rubidaea NCTC10036
S. plymuthica AS9
S. liquefaciens HUMV-21
S. grimesii NCTC11543
S. proteamaculans 4330
S. quinivorans 197
S. ficaria 5548
S. entomophila 8806213
S. marcescens Db11
S. marcescens 2880
10 kb
Conserved T6SS genes
Flanking genes

Figure S1. Representative Type VI secretion system gene clusters from *Serratia* including further information regarding the genomic context of i3v1 gene clusters. Related to Figure 1.

(A) Representive gene clusters of the different Type VI secretion system subtypes identified in *Serratia*. These are the same gene clusters as depicted in Figure 1B but also include the genomic identifiers for the first and last gene in each cluster. The T6SS subtype (i1, i2, i3v1, i3v2, i3v3 or i4b) is indicated on the left and genes encoding core T6SS components are coloured according to the key.

(B, C) Genomic context of representative T6SS_i3v1 gene clusters across *Serratia*. (B) Synteny plot of representative i3v1 T6SS gene clusters from each species of *Serratia* which encodes this T6SS subtype as shown in Figure 1C, but with an additional 30 kb on either side of the i3v1 T6SS genes. The synteny plot is displayed alongside a representative phylogenetic tree showing the relative position of the corresponding strain within the *Serratia* phylogeny, extracted from the tree in Figure 1A. Genes encoding core T6SS components are coloured according to the key, with arrows representing protein coding genes (CDSs) outlined in black and those representing tRNAs in red. Shading between representative gene clusters indicates pairwise percentage nucleotide identity as shown in the key. (C) Synteny plot of the same genomic region as shown in part B but with the inclusion of the three species which lack the i3v1 T6SS, *S. plymuthica*, *S. liquefaciens* and *S. grimesii*. Conserved T6SS genes are coloured black and the genes that flank the T6SS gene cluster or its inferred former location are arbitrarily coloured to indicate corresponding genes in different species. The differences in the remaining genes in the former location of the i3v1T6SS in the species in which it has been lost suggest that the loss has occurred on independent occasions.

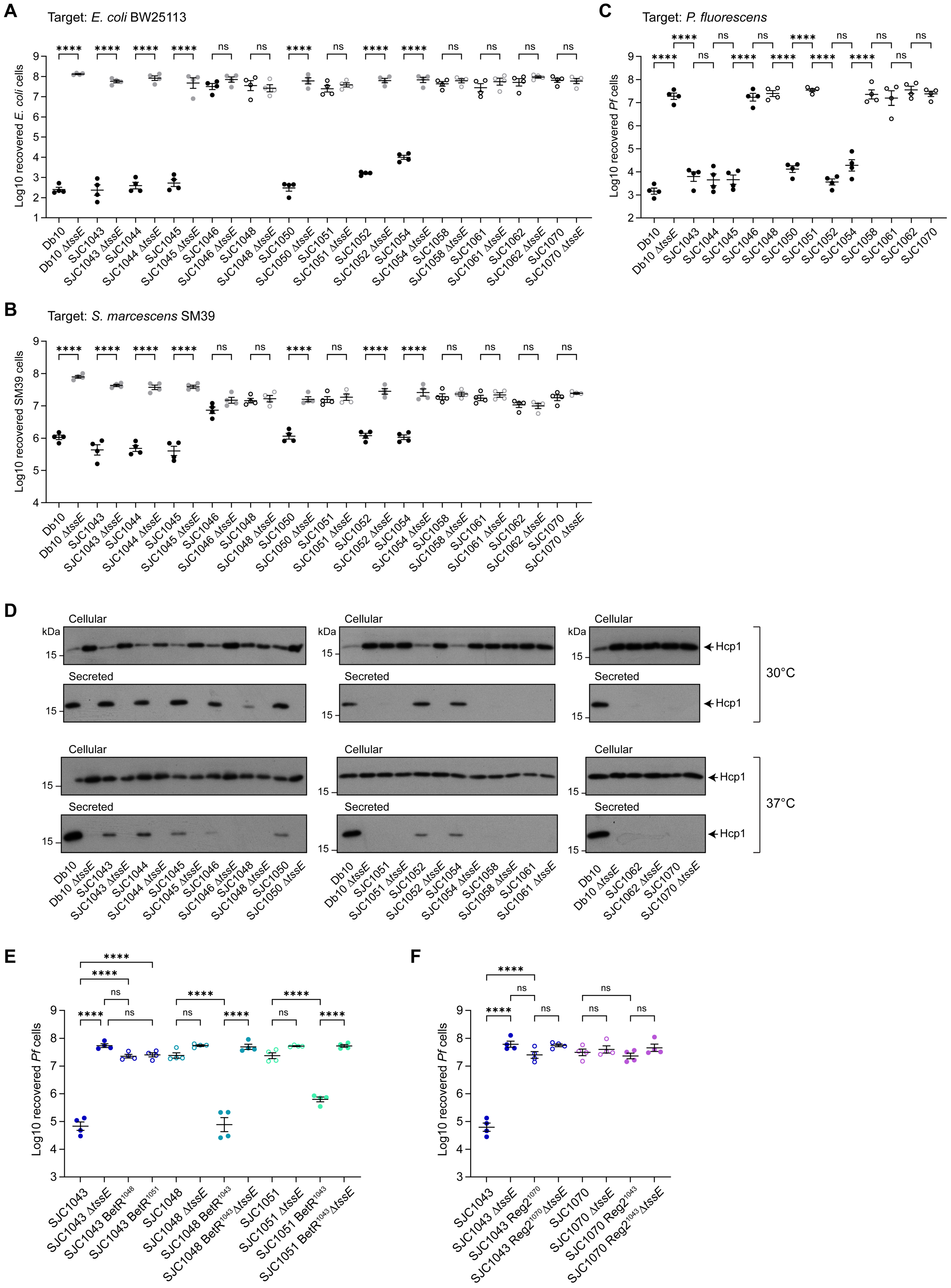
A
Target: E. coli BW25113
Log10 recovered E. coli cells
B
Target: S. marcescens SM39
Log10 recovered SM39 cells
Db10
Db10 ΔtssE
SJC1043
SJC1043 ΔtssE
SJC1044
SJC1044 ΔtssE
SJC1045
SJC1045 ΔtssE
SJC1046
SJC1046 ΔtssE
SJC1048
SJC1048 ΔtssE
SJC1050
SJC1050 ΔtssE
SJC1051
SJC1051 ΔtssE
SJC1052
SJC1052 ΔtssE
SJC1054
SJC1054 ΔtssE
SJC1058
SJC1058 ΔtssE
SJC1061
SJC1061 ΔtssE
SJC1062
SJC1062 ΔtssE
SJC1070
SJC1070 ΔtssE
C
Target: P. fluorescens
Log10 recovered Pf cells
D
Cellular
Secreted
kDa
15
Hcp1
30°C
37°C
E
Log10 recovered Pf cells
SJC1043 BetR^{1048}
SJC1043 BetR^{1051}
SJC1048 BetR^{1043}
SJC1048 BetR^{1043} ΔtssE
SJC1051 BetR^{1043}
SJC1051 BetR^{1043} ΔtssE
F
SJC1043 Reg2^{1070}
SJC1043 Reg2^{1070} ΔtssE
SJC1070 Reg2^{1043}
SJC1070 Reg2^{1043} ΔtssE
****
ns

Figure S2. Type VI secretion system-dependent antibacterial activity and Hcp secretion of clinical isolates of *Serratia marcescens* and antibacterial activity of strains with exchanged *betR* or *reg2* alleles against *P. fluorescens*. Related to Figure 2.

(A-C) Type VI secretion system-dependent antibacterial activity of clinical isolates of *Serratia marcescens*. Recovery of target organisms (A) *E. coli* BW25113, (B) *S. marcescens* SM39, or (C) *P. fluorescens*, following 4 hr co-culture with wild type *S. marcescens* Db10 (Db10), wild type clinical isolates of *S. marcescens* (SJC1043-SJC1070) or the corresponding T6SS-inactive Δ*tssE* mutants, as indicated, at an initial ratio of 1:1

(D) Hcp secretion by clinical isolates of *Serratia marcescens*. Immunoblot detection of Hcp1 (from T6SS_i3v1) in cellular and secreted fractions of wild type *S. marcescens* Db10 (Db10), wild type clinical isolates of *S. marcescens* (SJC1043-SJC1070), or the corresponding T6SS_i3v1-inactive Δ*tssE* mutants, as indicated, when cultures were grown at 30°C or 37°C as indicated. The data are representative of two independent experiments.

(E, F) Type VI secretion system-dependent antibacterial activity of strains with exchanged *betR* or *reg2* alleles against *P. fluorescens*. Recovery of target *P. fluorescens* cells following co-culture with wild type clinical isolates of *S. marcescens* or derivatives with an exchanged *betR* (E) or *reg2* (F) allele, as indicated. SJC1043 BetR^{1048} indicates SJC1043 with the *betR* allele from SJC1048, and similarly for the other exchanges.

For antibacterial activity assays, data are presented as mean ± SEM with individual data points overlaid (n=4 biological replicates; **** P<0.0001, ns not significant; one-way ANOVA with Tukey's test; for clarity, only selected comparisons are displayed). Filled circles indicate isolates with *betR* and *reg2* intact, open circles indicate isolates with *betR* or *reg2* disrupted.

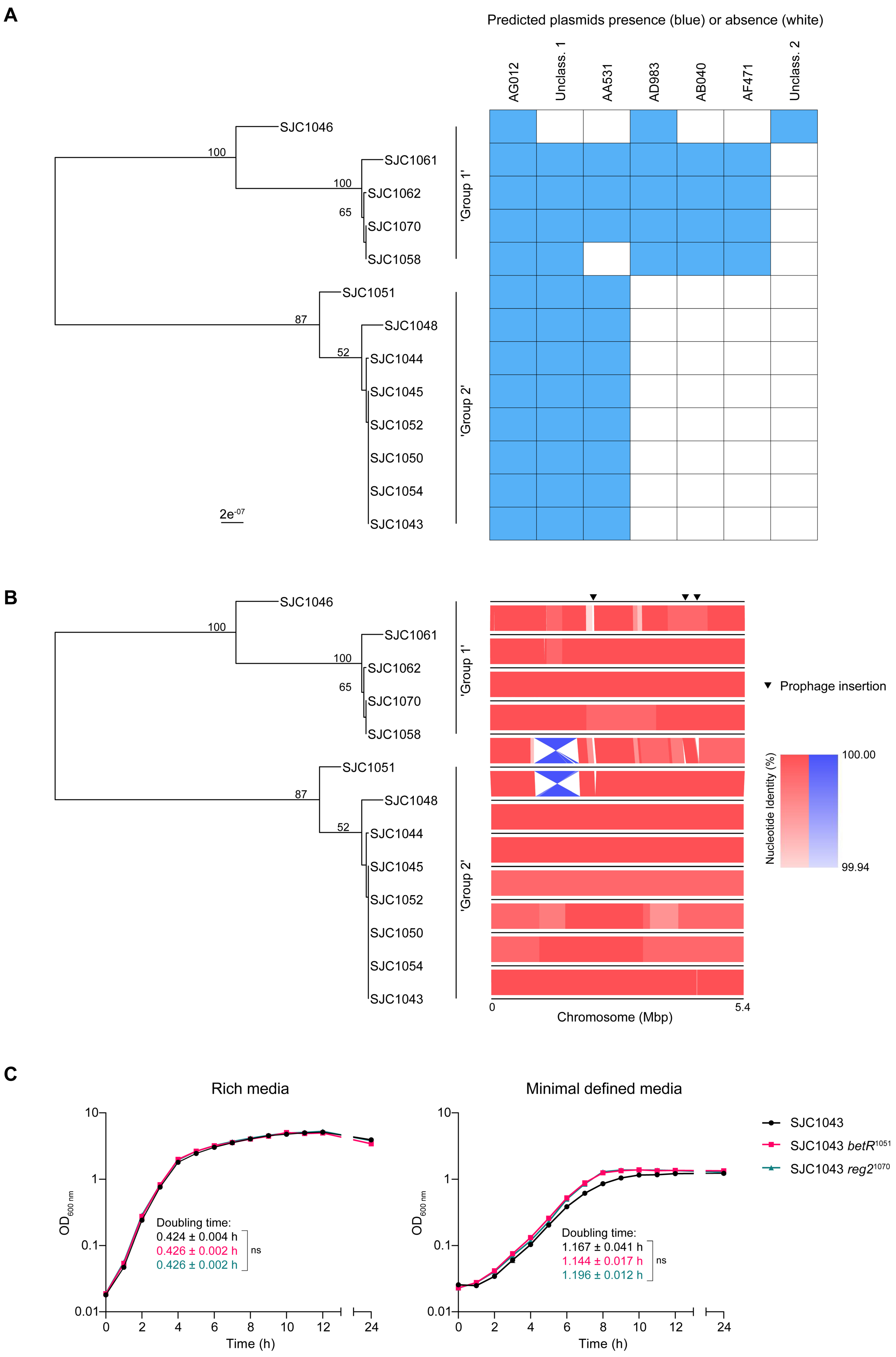

A
Predicted plasmids presence (blue) or absence (white)
AG012
Unclass. 1
AA531
AD983
AB040
AF471
Unclass. 2
SJC1046
SJC1061
SJC1062
SJC1070
SJC1058
SJC1051
SJC1048
SJC1044
SJC1045
SJC1052
SJC1050
SJC1054
SJC1043
100
100
65
87
52
'Group 1'
'Group 2'
2e-07
B
Prophage insertion
Nucleotide Identity (%)
100.00
99.94
0
5.4
Chromosome (Mbp)
C
Rich media
Minimal defined media
Doubling time:
0.424 ± 0.004 h
0.426 ± 0.002 h
0.426 ± 0.002 h
ns
Doubling time:
1.167 ± 0.041 h
1.144 ± 0.017 h
1.196 ± 0.012 h
ns
OD600 nm
Time (h)
SJC1043
SJC1043 betR1051
SJC1043 reg21070

Figure S3. Plasmid occurrence, chromosomal synteny and growth of closely-related isolates of *Serratia marcescens* with intact and disrupted *betR* and *reg2* alleles. Related to Figure 2.

(A, B) Presence/absence of plasmids and chromosomal synteny in a group of closely-related isolates of *Serratia marcescens*, in each case plotted alongside the phylogenetic tree shown in Figure 2A. (A) Extra-chromosomal circular DNA regions were grouped by clustering using Mash. Groups are named according to typed plasmid groups in Williams *et al*., 2022, or as unclassified putative novel plasmids (Unclass.) if they did not match typed groups in this previous study but showed similarity (>40% blastn coverage) to previously identified plasmids. (B) Synteny of complete chromosomes. Blocks of colour represent homologous regions in the same (red) or opposite (blue) orientation, with the depth of the colour corresponding to pairwise percentage nucleotide identity determined using blastn as indicated by the key.

(C) Growth of *S. marcescens* SJC1043 with wild type and disrupted *betR* and *reg2* genes at 37°C, 200 rpm in 25 ml rich media (LB, left) or minimal defined media (MM, right), measured as optical density of the culture at 600 nm (OD_{600nm}) over time. Points show mean ± SEM; error bars smaller than the symbols are not plotted. Mean doubling (generation) time during exponential growth +/- SEM is shown for each strain; doubling time was not significantly different between strains (one-way ANOVA with Tukey's test).

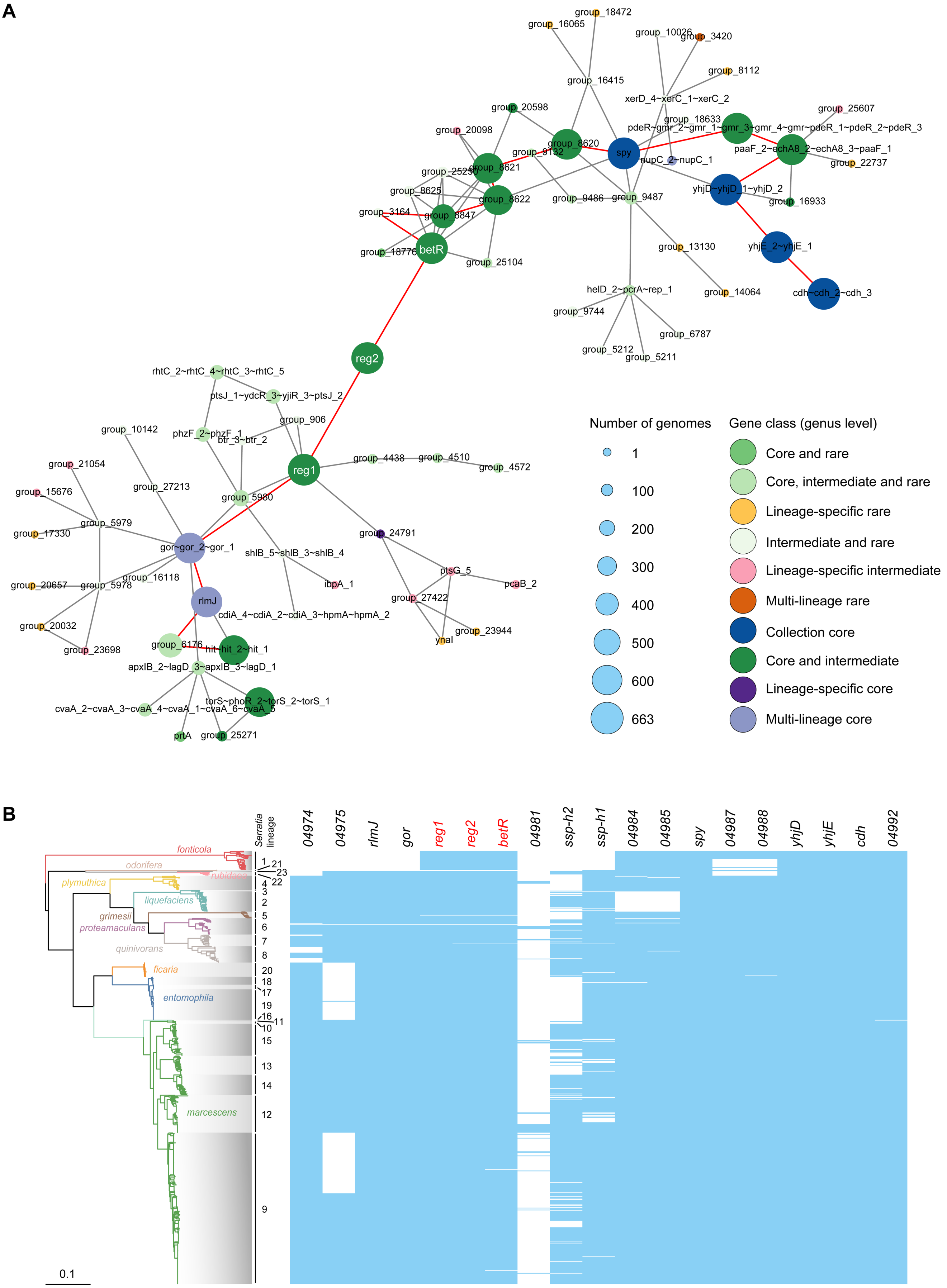
A
B
betR
reg1
reg2
rlmJ
spy
Number of genomes
1
100
200
300
400
500
600
663
Gene class (genus level)
Core and rare
Core, intermediate and rare
Lineage-specific rare
Intermediate and rare
Lineage-specific intermediate
Multi-lineage rare
Collection core
Core and intermediate
Lineage-specific core
Multi-lineage core
Serratia lineage
04974
04975
rlmJ
gor
reg1
reg2
betR
04981
ssp-h2
ssp-h1
04984
04985
spy
04987
04988
yhjD
yhjE
cdh
04992
fonticola
odorifera
rubidaea
plymuthica
liquefaciens
grimesii
proteamaculans
quinivorans
ficaria
entomophila
marcescens
0.1

Figure S4. Conservation of *betR* across *Serratia*. Related to Figure 3.

(A) Subsection of the *Serratia* pan-genome network, centred on *betR*. Gene groups were selected by taking *betR* and the iterative six nearest undirected neighbouring nodes. Red line represents the gene path found in *S. marcescens* SJC1043. Node size corresponds to the number of genomes in which the gene group is found. Node colour corresponds to the conservation level of the gene group across the genus. Note that the colouring here is different to that in Figure 3 because annotations for *reg1*, *reg2* and *betR* are missing in a small number of genomes across the genus (see panel B), resulting in these gene groups not being classed as collection core for the genus. The 'missing' annotations are partly as a result of Reg1, Reg2 and BetR in two out of the three *S. odorifera* genomes in the dataset forming separate protein groups to the corresponding proteins across the rest of the genus. Additionally, annotations for *reg1*, *reg2* and *betR* in two *S. marcescens* genomes and in individual *S. quinivorans*, *S. grimesii*, *S. proteamaculans* and *S. odorifera* genomes are altered or missing due to contig breaks,insertions or large insertions.

(B) Presence/absence across *Serratia* of the genes upstream, downstream, and within the *betR* locus of *S. marcescens* SJC1043.

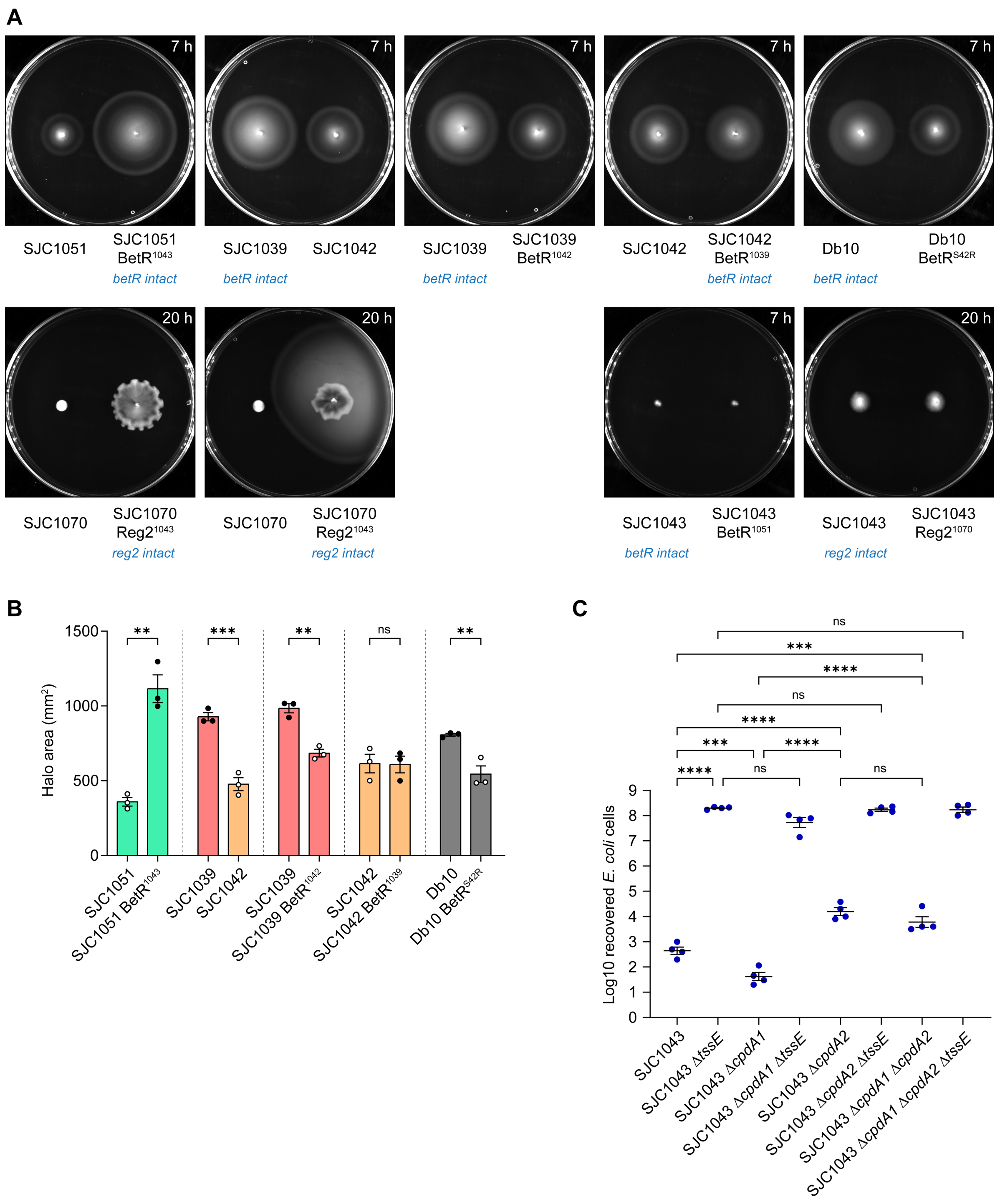

Figure S5. Phenotypic assays of *betR*-dependent traits, particularly swimming motility. Related to Figure 6.

(A, B) Intact *betR* and *reg2* promote motility in *S. marcescens*. Swimming motility in 0.3% semisolid agar over 7 h was compared between wild type *S. marcescens* SJC1051 (*betR* disrupted) and SJC1051 $BetR^{1043}$ (intact *betR* restored), SJC1039 (*betR* intact) and SJC1042 (*betR* disrupted), SJC1039 (*betR* intact) and SJC1039 $BetR^{1042}$ (*betR* disrupted), SJC1042 (*betR* disrupted) and SJC1042 $BetR^{1039}$ (intact *betR* restored), and Db10 (*betR* intact) and Db10 $BetR^{S42R}$ (functionally-impaired BetR point mutant). Wild type *S. marcescens* SJC1070 (*betR* disrupted) did not display any motility in 0.3% semisolid agar, but SJC1070 $BetR^{1043}$ (intact *betR* restored) displayed variable swarming- or swimming-like behaviours over 20 h. Wild type *S. marcescens* SJC1043 (*betR* intact) and its

derivatives did not display swimming (or swarming) behaviour under the conditions tested, for reasons that are currently unknown. (A) Example images of swimming motility plates. (B) Quantification of motility halo area over 7 h for the isolates which displayed classical swimming behaviour. Bars show mean ± SEM with individual data points overlaid (n=3 biological replicates; *** $P<0.001$, ** $P<0.01$; students T-test between strains compared directly).

(C) Type VI secretion system-dependent antibacterial activity of Δ*cpdA1* and Δ*cpdA2* mutants of *Serratia marcescens* SJC1043. Recovery of *E. coli* BW25113 target cells following 4 hr co-culture with wild type *S. marcescens* SJC1043 or mutants carrying the in-frame deletions Δ*cpdA1*, Δ*cpdA2* and/or Δ*tssE*, as indicated, at an initial ratio of 1:1. The Δ*cpdA1* and Δ*cpdA2* mutants lack one of the genes encoding cAMP phosphodiesterase enzymes; Δ*tssE* mutants have an inactive T6SS, confirming that antibacterial activity observed in the *cpdA* mutants is T6SS dependent. Data are presented as mean ± SEM with individual data points overlaid (n=4 biological replicates; **** $P<0.0001$, *** $P<0.001$, ns not significant; one-way ANOVA with Tukey's test; for clarity, only selected comparisons are displayed).

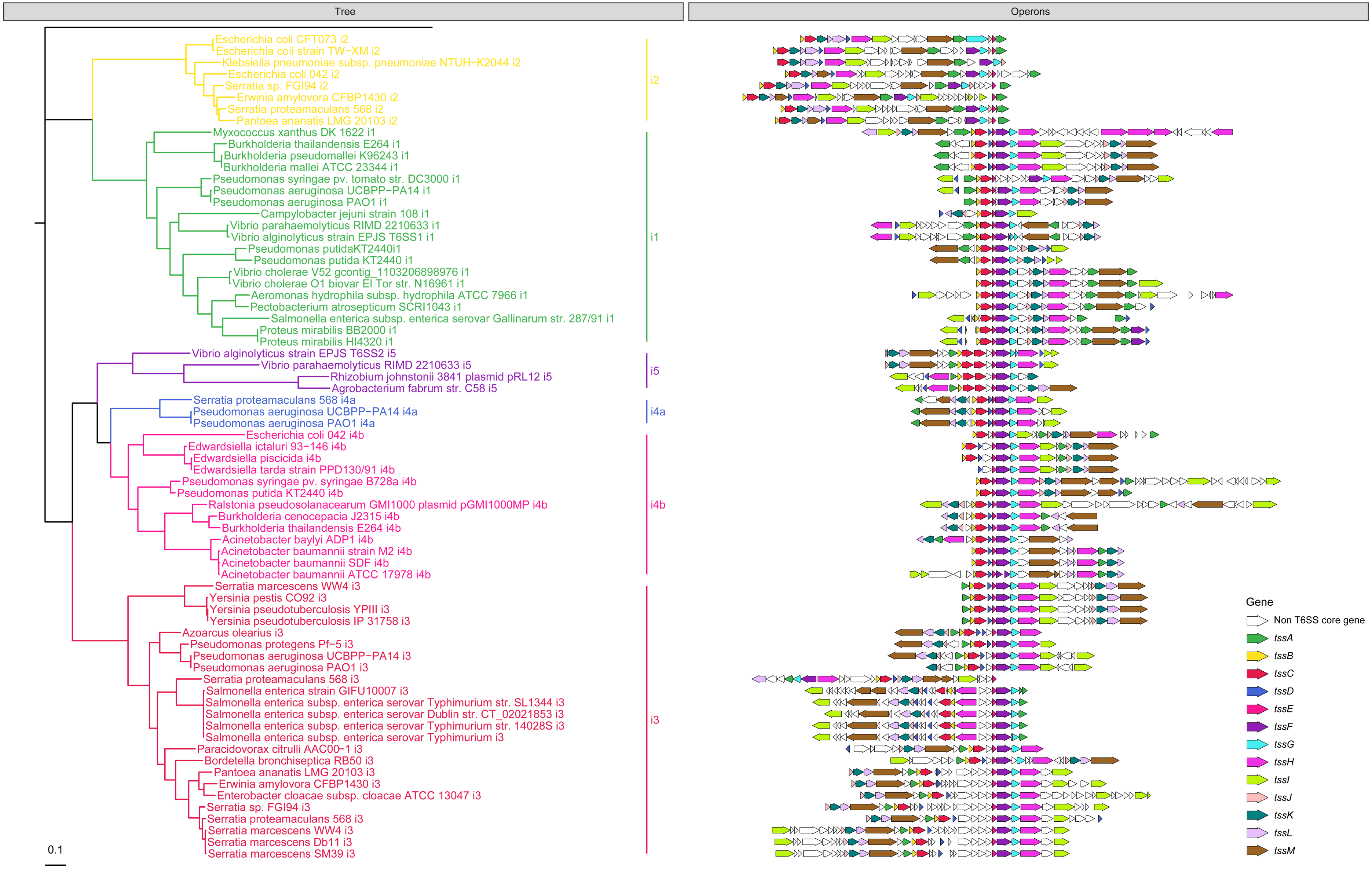

Tree
Operons
Escherichia coli CFT073 i2
Escherichia coli strain TW−XM i2
Klebsiella pneumoniae subsp. pneumoniae NTUH−K2044 i2
Escherichia coli 042 i2
Serratia sp. FGI94 i2
Erwinia amylovora CFBP1430 i2
Serratia proteamaculans 568 i2
Pantoea ananatis LMG 20103 i2
Myxococcus xanthus DK 1622 i1
Burkholderia thailandensis E264 i1
Burkholderia pseudomallei K96243 i1
Burkholderia mallei ATCC 23344 i1
Pseudomonas syringae pv. tomato str. DC3000 i1
Pseudomonas aeruginosa UCBPP−PA14 i1
Pseudomonas aeruginosa PAO1 i1
Campylobacter jejuni strain 108 i1
Vibrio parahaemolyticus RIMD 2210633 i1
Vibrio alginolyticus strain EPJS T6SS1 i1
Pseudomonas putidaKT2440i1
Pseudomonas putida KT2440 i1
Vibrio cholerae V52 gcontig_1103206898976 i1
Vibrio cholerae O1 biovar El Tor str. N16961 i1
Aeromonas hydrophila subsp. hydrophila ATCC 7966 i1
Pectobacterium atrosepticum SCRI1043 i1
Salmonella enterica subsp. enterica serovar Gallinarum str. 287/91 i1
Proteus mirabilis BB2000 i1
Proteus mirabilis HI4320 i1
Vibrio alginolyticus strain EPJS T6SS2 i5
Vibrio parahaemolyticus RIMD 2210633 i5
Rhizobium johnstonii 3841 plasmid pRL12 i5
Agrobacterium fabrum str. C58 i5
Serratia proteamaculans 568 i4a
Pseudomonas aeruginosa UCBPP−PA14 i4a
Pseudomonas aeruginosa PAO1 i4a
Escherichia coli 042 i4b
Edwardsiella ictaluri 93−146 i4b
Edwardsiella piscicida i4b
Edwardsiella tarda strain PPD130/91 i4b
Pseudomonas syringae pv. syringae B728a i4b
Pseudomonas putida KT2440 i4b
Ralstonia pseudosolanacearum GMI1000 plasmid pGMI1000MP i4b
Burkholderia cenocepacia J2315 i4b
Burkholderia thailandensis E264 i4b
Acinetobacter baylyi ADP1 i4b
Acinetobacter baumannii strain M2 i4b
Acinetobacter baumannii SDF i4b
Acinetobacter baumannii ATCC 17978 i4b
Serratia marcescens WW4 i3
Yersinia pestis CO92 i3
Yersinia pseudotuberculosis YPIII i3
Yersinia pseudotuberculosis IP 31758 i3
Azoarcus olearius i3
Pseudomonas protegens Pf−5 i3
Pseudomonas aeruginosa UCBPP−PA14 i3
Pseudomonas aeruginosa PAO1 i3
Serratia proteamaculans 568 i3
Salmonella enterica strain GIFU10007 i3
Salmonella enterica subsp. enterica serovar Typhimurium str. SL1344 i3
Salmonella enterica subsp. enterica serovar Dublin str. CT_02021853 i3
Salmonella enterica subsp. enterica serovar Typhimurium str. 14028S i3
Salmonella enterica subsp. enterica serovar Typhimurium i3
Paracidovorax citrulli AAC00−1 i3
Bordetella bronchiseptica RB50 i3
Pantoea ananatis LMG 20103 i3
Erwinia amylovora CFBP1430 i3
Enterobacter cloacae subsp. cloacae ATCC 13047 i3
Serratia sp. FGI94 i3
Serratia proteamaculans 568 i3
Serratia marcescens WW4 i3
Serratia marcescens Db11 i3
Serratia marcescens SM39 i3
0.1
i2
i1
i5
i4a
i4b
i3
Gene
Non T6SS core gene
tssA
tssB
tssC
tssD
tssE
tssF
tssG
tssH
tssI
tssJ
tssK
tssL
tssM

Figure S6. Phylogenetic structure of reference set of T6SSs. Related to STAR Methods and Table S5.

Maximum-likelihood tree of aligned and concatenated TssB and TssC sequences from a set of experimentally-characterised and manually-curated predicted T6SSs, representing the reference set of T6SSs for this study (details in Table S5). T6SS clades are coloured according to subtype (i1-i5)[S1]. Gene organisation is shown, to scale, alongside each tip, aligned to *tssE*. The outgroup is the type iii T6SS from *Bacteroides fragilis* NCTC 9343.

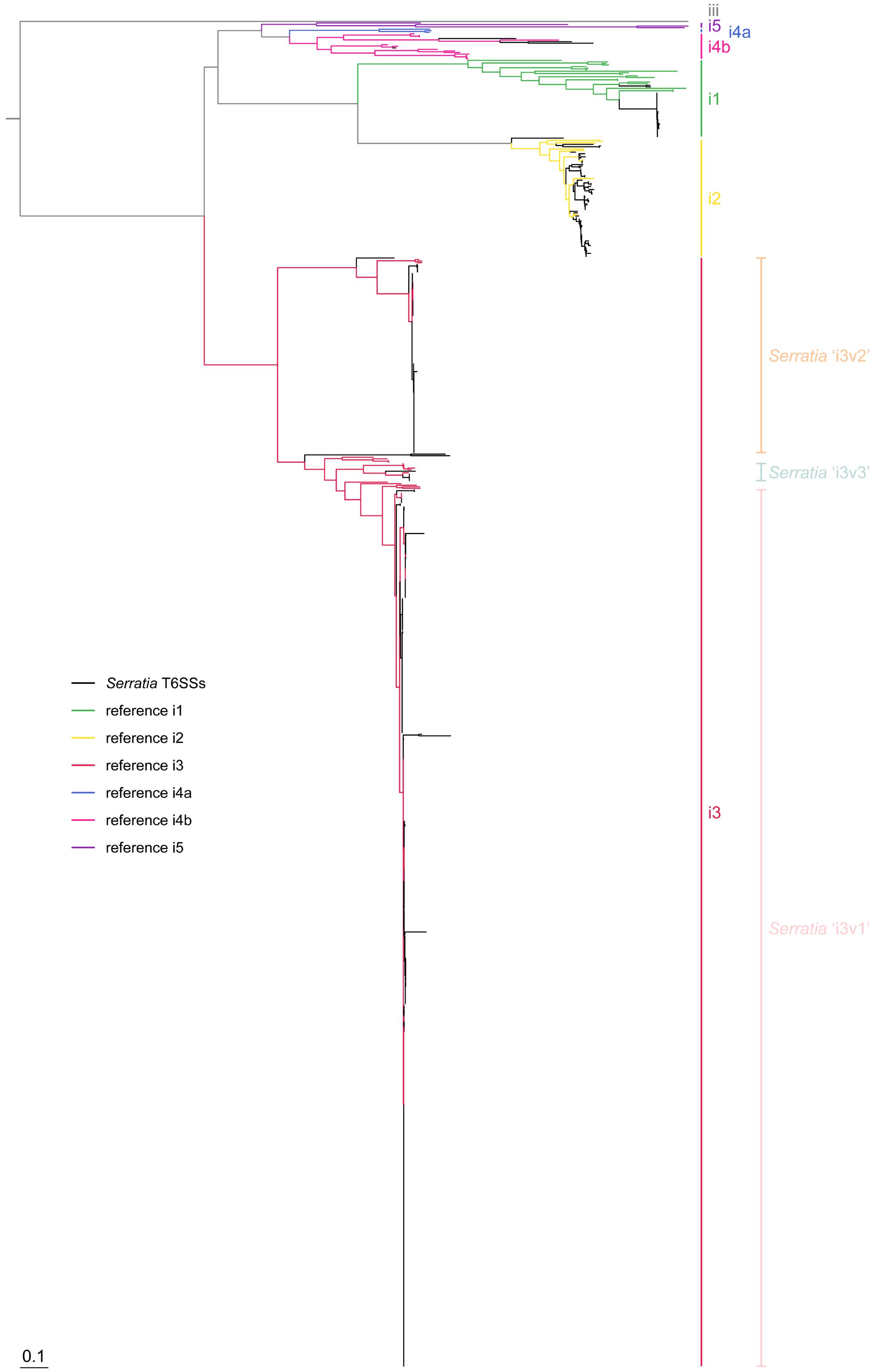

Figure S7. Subtyping of predicted *Serratia* T6SSs. Related to Figure 1 and STAR Methods. Maximum-likelihood tree of aligned and concatenated TssB and TssC sequences from reference T6SSs and predicted *Serratia* T6SSs. Tips corresponding to T6SSs from the reference set are coloured according to subtype (i1-i5). Tips corresponding to T6SSs predicted from the *Serratia* dataset are coloured black. Clades corresponding to within-subtype groups identified in *Serratia* (i3v1-i3v3) are shown on the right.

Table S1. Occurrence of Type VI secretion system subtypes across the genus *Serratia*. Related to Figure 1.

Species	Lineage	Number of genomes	Number of genomes with T6SS of subtype							
			i1	i2	i3v1	i3v2	i3v3	i3 other	i4b	Und.
Serratia fonticola	1	29	29	0	2	0	0	0	2	30
Serratia liquefaciens	2	31	0	0	0	0	0	0	0	0
Serratia liquefaciens	3	2	0	1	0	0	0	0	0	0
Serratia plymuthica	4	22	0	6	0	0	6	0	0	0
Serratia grimesii	5	10	0	0	0	0	0	1	0	0
Serr. proteamaculans	6	25	0	8	17	6	0	1	0	3
Serratia quinivorans	7	18	0	21	16	0	0	0	0	8
Serratia quinivorans	8	25	0	0	25	0	0	0	0	1
Serratia marcescens	9	232	0	0	232	0	0	0	0	6
Serratia marcescens	10	4	0	0	4	4	0	0	0	3
Serratia marcescens	11	1	0	0	1	1	0	0	0	0
Serratia marcescens	12	58	0	3	57	53	0	0	0	4
Serratia marcescens	13	29	0	6	29	0	0	0	0	2
Serratia marcescens	14	31	1	0	31	30	0	0	0	4
Serratia marcescens	15	49	1	2	49	27	0	0	1	6
Serr. marcescens-like	16	1	0	2	0	0	0	0	0	0
Serratia entomophila	17	7	0	3	7	0	0	0	0	0
Serratia entomophila	18	12	0	0	12	0	0	0	0	0
Serratia entomophila	19	47	0	0	47	0	0	0	0	0
Serratia ficaria	20	22	0	10	21	1	0	0	1	1
Serratia odorifera	21	2	0	0	2	0	0	0	0	0
Serratia rubidaea	22	6	0	5	6	0	0	0	0	1
Serratia rubidaea-like	23	1	0	1	1	0	0	0	0	1

Genomic Identifier	Gene name	SJC1043 / SJC1043 BetR^{1051}		SJC1051 BetR^{1043} / SJC1051		SJC1043 / SJC1043 Reg2^{1070}		Known or predicted product	Gene cluster/group	Conservation
		Log2 FC	adj. p val	Log2 FC	adj. p val	Log2 FC	adj. p val			
SJC1043_00094		**1.630**	**1.68E-14**	0.781	8.27E-02	**1.178**	**2.22E-08**	NAD-dependent epimerase/dehydratase for sugar-nucleotide substrates		Collection core
SJC1043_00176	glpQ	**-1.924**	**3.24E-13**	-0.684	2.13E-01	**-1.629**	**5.41E-12**	Glycerophosphodiester phosphodiesterase	Glycerol uptake and metabolism	Collection core
SJC1043_00177	glpT	**-1.795**	**8.05E-06**	-1.141	1.36E-01	**-1.367**	**5.64E-04**	Glycerol 3-phosphate uptake transporter	Glycerol uptake and metabolism	Collection core
SJC1043_00178	glpA	**-1.511**	**8.43E-06**	-0.602	8.49E-01	**-1.214**	**1.42E-04**	Glycerol-3-phosphate dehydrogenase subunit A	Glycerol uptake and metabolism	Collection core
SJC1043_00179	glpB	**-1.875**	**1.14E-05**	-0.564	8.79E-01	-1.214	1.63E-02	Glycerol-3-phosphate dehydrogenase subunit B	Glycerol uptake and metabolism	Collection core
SJC1043_00180	glpC	**-1.665**	**7.75E-03**	-0.826	4.31E-01	-0.954	2.10E-01	Glycerol-3-phosphate dehydrogenase subunit C	Glycerol uptake and metabolism	Collection core
SJC1043_00214	metE	0.897	6.77E-01	**2.503**	**1.54E-16**	-0.363	8.96E-01	Cobalamin-independent methionine synthase		Collection core
SJC1043_00375	tsr_1	**1.807**	**3.66E-03**	**1.936**	**5.62E-03**	1.241	1.11E-01	Methyl-accepting chemotaxis protein	Flagellar	Collection core
SJC1043_00512		**3.645**	**1.01E-19**	**2.756**	**2.22E-12**	**2.372**	**1.50E-07**	Coenzyme PQQ synthesis protein D	Phage cargo?	Lineage specific intermediate
SJC1043_00513		**2.653**	**1.27E-08**	**1.987**	**1.86E-04**	**2.162**	**3.34E-11**	S-adenosylmethionine-dependent methyltransferase	Phage cargo?	Lineage specific intermediate
SJC1043_00514		**3.468**	**1.97E-18**	**2.536**	**2.66E-08**	**2.297**	**4.12E-10**	Major facilitator superfamily membrane transporter	Phage cargo?	Lineage specific intermediate
SJC1043_00515		**2.861**	**5.34E-16**	**1.595**	**4.53E-03**	**1.961**	**1.26E-09**	Cupin-like metalloenzyme	Phage cargo?	Lineage specific intermediate
SJC1043_00516		**1.670**	**5.79E-10**	**1.369**	**9.62E-04**	0.996	8.27E-03	Glutamine amidotransferase (asparagine synthase-like)	Phage cargo?	Lineage specific intermediate
SJC1043_00520		0.673	1.86E-01	0.016	1.00E+00	**1.032**	**9.83E-03**	Protein of unknown function		Multi-lineage intermediate
SJC1043_00527	mrr	**-1.366**	**1.39E-03**	0.188	9.95E-01	**-1.498**	**1.60E-16**	Mrr, type IV restriction endonuclease		Core, intermediate and rare
SJC1043_00531		0.781	5.58E-01	0.063	9.97E-01	**1.632**	**3.20E-04**	Protein of unknown function		Multi-lineage intermediate
SJC1043_00550		-0.731	1.00E-02	0.716	3.29E-01	**-1.144**	**5.62E-11**	Protein of unknown function		Collection intermediate
SJC1043_00711	sgrT	**-1.542**	**1.64E-03**	0.404	9.30E-01	-0.870	1.89E-01	Inhibitor of glucose uptake transporter		Collection core
SJC1043_00786	cysC	**1.173**	**2.44E-03**	0.611	2.93E-01	**1.004**	**2.03E-04**	Adenylyl-sulfate kinase		Collection core
SJC1043_00787	ygbE	0.336	5.77E-01	**1.204**	**3.10E-04**	0.033	9.75E-01	Inner membrane protein (contains DUF3561)		Collection core
SJC1043_00840		**1.327**	**3.06E-03**	-0.097	9.95E-01	**1.219**	**8.52E-03**	Non-coding RNA (Phe leader)		NA
SJC1043_00869	mtnB	**1.597**	**3.46E-03**	0.436	5.91E-01	1.069	8.41E-02	Methylthioribulose-1- phosphate dehydratase		Collection core
SJC1043_00985		0.614	1.57E-01	**1.188**	**3.48E-04**	0.390	3.34E-01	Protein of unknown function	Prophage	Multi-lineage intermediate
SJC1043_00986		0.618	5.51E-02	**1.113**	**8.86E-05**	0.223	6.51E-01	Protein of unknown function	Prophage	Lineage specific intermediate
SJC1043_00987		0.727	6.06E-02	**1.120**	**7.22E-06**	0.287	5.73E-01	Protein of unknown function	Prophage	Lineage specific rare
SJC1043_00988	exo	0.869	9.58E-03	**1.156**	**1.98E-05**	0.426	3.54E-01	YqaJ-like viral recombinase, phage endonuclease	Prophage	Lineage specific intermediate
SJC1043_00989	recT	0.844	2.71E-03	**1.216**	**1.11E-08**	0.529	8.45E-02	Recombinational DNA repair protein	Prophage	Intermediate and rare
SJC1043_00990		**1.042**	**8.69E-06**	**1.271**	**1.20E-06**	0.758	6.40E-03	Protein of unknown function	Prophage	Intermediate and rare
SJC1043_00992		0.487	1.73E-01	**1.304**	**2.48E-06**	0.197	5.65E-01	Protein of unknown function	Prophage	Lineage specific rare
SJC1043_00993		0.730	7.77E-03	**1.207**	**9.77E-08**	0.399	2.24E-01	Protein of unknown function	Prophage	Lineage specific intermediate
SJC1043_00997		0.807	3.00E-03	**1.069**	**5.13E-05**	0.383	2.93E-01	Bacteriophage replication protein O	Prophage	Lineage specific intermediate
SJC1043_00999		0.770	1.93E-02	**1.079**	**3.24E-03**	0.422	3.11E-01	Protein of unknown function	Prophage	Lineage specific rare
SJC1043_01001		0.838	8.10E-02	**1.241**	**1.57E-03**	0.502	3.11E-01	Protein of unknown function	Prophage	Lineage specific intermediate
SJC1043_01002	ninB	0.628	1.52E-01	**1.111**	**2.25E-03**	0.446	1.82E-01	NinB protein	Prophage	Intermediate and rare
SJC1043_01072		**-1.335**	**4.29E-04**	-0.024	9.98E-01	-1.021	2.06E-02	Helix-hairpin-helix DNA-binding protein		Collection core
SJC1043_01104	acrR	-0.972	5.59E-03	-0.313	8.79E-01	**-1.114**	**2.12E-05**	Transcriptional repressor of the acrAB operon		Collection core
SJC1043_01150	ybbO	**-1.786**	**9.56E-05**	0.546	5.79E-01	-0.772	1.71E-02	Short-chain dehydrogenase/reductase		Collection core
SJC1043_01284	chiC	0.730	3.46E-01	**3.142**	**1.44E-05**	0.676	1.10E-01	Secreted chitinase		Collection core
SJC1043_01420		**1.898**	**3.08E-03**	1.200	1.77E-01	1.106	2.09E-01	Membrane transport protein		Multi-lineage core
SJC1043_01693		**1.190**	**3.80E-03**	-0.072	9.95E-01	0.935	4.72E-02	Non-coding RNA (His leader)		NA
SJC1043_01711	sdaB	**-1.101**	**5.52E-04**	0.237	9.55E-01	-0.541	1.05E-01	L-serine deaminase II		Collection core
SJC1043_01727		**1.069**	**4.29E-04**	0.402	7.37E-01	0.764	2.47E-02	Ankyrin repeat domain containing protein		Intermediate and rare
SJC1043_01978	msyB	**1.055**	**9.10E-05**	0.127	9.94E-01	0.918	4.38E-03	MsyB protein		Collection core
SJC1043_02192	puuB	**1.841**	**3.87E-06**	0.224	9.95E-01	**1.070**	**4.65E-03**	γ-Glutamylputrescine oxidase	Putrescine uptake and utilisation	Collection core
SJC1043_02194	puuR	**1.979**	**7.87E-03**	0.126	9.98E-01	1.226	3.87E-02	Transcriptional regulator	Putrescine uptake and utilisation	Collection core
SJC1043_02195	puuD	**2.248**	**1.34E-03**	0.227	9.95E-01	**1.684**	**1.62E-03**	γ-Glutamyl-γ-aminobutyrate hydrolase	Putrescine uptake and utilisation	Collection core
SJC1043_02196	puuA	**2.956**	**4.35E-03**	-0.229	9.95E-01	1.923	1.37E-02	Glutamate-putrescine ligase	Putrescine uptake and utilisation	Core and intermediate
SJC1043_02197	puuP	**2.887**	**2.70E-07**	-0.082	9.98E-01	**1.634**	**2.14E-03**	Putrescine transporter	Putrescine uptake and utilisation	Collection core
SJC1043_02356	sodC	**1.262**	**3.92E-06**	0.811	1.08E-01	0.767	4.05E-02	Cu-Zn superoxide dismutase		Collection core

SJC1043_02465		**-1.294**	**7.96E-04**	0.666	3.47E-01	-0.407	4.88E-01	Chromate transporter		Collection core
SJC1043_02472	eagR2	**1.095**	**9.14E-07**	1.096	3.18E-02	0.191	7.64E-01	PAAR chaperone protein		Collection core
SJC1043_02474		**1.265**	**6.44E-03**	0.609	7.44E-01	0.873	3.86E-02	Protein of unknown function		Intermediate and rare
SJC1043_02476		**1.042**	**1.66E-03**	0.619	3.55E-01	0.714	8.94E-02	Protein of unknown function		Intermediate and rare
SJC1043_02697		1.182	1.54E-02	**1.601**	**3.24E-05**	-0.116	9.35E-01	Protein of unknown function		Collection core
SJC1043_02990		**-1.163**	**5.01E-05**	-0.817	1.14E-01	-0.762	6.75E-03	AriR-like regulator, may regulate biofilm formation		Core and intermediate
SJC1043_03064	fliZ	1.490	8.02E-02	**2.020**	**1.08E-04**	0.884	3.79E-01	Transcriptional regulator	Flagellar	Collection core
SJC1043_03066	fliC	**2.592**	**2.94E-04**	**2.552**	**8.78E-04**	0.332	9.15E-01	Flagellin; flagellar filament structural protein	Flagellar	Core and intermediate
SJC1043_03067	fliD	**2.567**	**3.80E-03**	**2.413**	**6.68E-03**	1.255	4.93E-01	Flagellar filament capping protein	Flagellar	Core and intermediate
SJC1043_03068	fliS	**2.315**	**8.29E-03**	1.720	2.13E-01	1.241	3.31E-01	Flagellar chaperone protein	Flagellar	Core and intermediate
SJC1043_03069	fliT	**2.406**	**4.29E-03**	**2.506**	**3.79E-03**	1.465	2.17E-01	Flagellar chaperone protein	Flagellar	Core and intermediate
SJC1043_03071		**1.330**	**1.27E-03**	0.884	1.43E-02	0.768	2.06E-01	Small protein of unknown function	Flagellar	Core and intermediate
SJC1043_03099	flgE	1.277	1.22E-01	**1.716**	**2.11E-03**	1.124	1.55E-01	Flagellar hook protein	Flagellar	Collection core
SJC1043_03100	flgD	1.581	2.28E-01	**2.116**	**1.94E-04**	1.060	3.71E-01	Flagellar hook assembly protein	Flagellar	Collection core
SJC1043_03119	cheW	**2.624**	**2.71E-03**	1.964	4.14E-01	1.086	6.32E-01	Chemotaxis protein	Flagellar	Collection core
SJC1043_03121	motB	**2.585**	**1.37E-03**	2.475	9.03E-02	1.327	5.06E-01	Stator unit of the flagellar motor complex (with MotB)	Flagellar	Collection core
SJC1043_03122	motA	**2.656**	**1.71E-03**	2.168	4.53E-01	1.414	5.34E-01	Stator unit of the flagellar motor complex (with MotA)	Flagellar	Collection core
SJC1043_03123	flhC	**1.975**	**3.64E-06**	1.237	8.98E-02	**1.427**	**4.65E-03**	Flagellar transcriptional regulator (with FlhD)	Flagellar	Collection core
SJC1043_03124	flhD	**1.257**	**3.18E-03**	**1.199**	**1.22E-03**	0.646	2.06E-01	Flagellar transcriptional regulator (with FlhC)	Flagellar	Collection core
SJC1043_03171	alb6	**1.781**	**7.75E-05**	**1.662**	**2.12E-04**	**1.473**	**7.25E-03**	Thioesterase	Althiomycin NRPS-PKS	Core and intermediate
SJC1043_03172	alb5	**2.418**	**6.78E-09**	**2.286**	**1.67E-10**	**1.658**	**2.03E-04**	Hybrid non-ribosomal peptide synthetase/ polyketide synthase	Althiomycin NRPS-PKS	Core and intermediate
SJC1043_03173	alb4	**2.677**	**1.22E-14**	**2.762**	**8.37E-25**	**1.845**	**1.65E-15**	Non-ribosomal peptide synthetase	Althiomycin NRPS-PKS	Core and intermediate
SJC1043_03174	alb3	**3.920**	**5.69E-10**	**4.662**	**8.31E-40**	**2.110**	**2.60E-12**	O-Methyltransferase	Althiomycin NRPS-PKS	Core and intermediate
SJC1043_03175	alb2	**4.487**	**2.82E-91**	**5.612**	**1.44E-71**	**1.803**	**1.04E-04**	Fe/Mn-dependent N-oxygenase	Althiomycin NRPS-PKS	Core and intermediate
SJC1043_03176	alb1	**4.139**	**2.54E-131**	**4.252**	**1.57E-75**	**1.955**	**3.59E-13**	Major facilitator superfamily althiomycin efflux pump	Althiomycin NRPS-PKS	Core and intermediate
SJC1043_03183		**1.059**	**1.73E-03**	0.743	3.21E-01	0.411	4.06E-01	Protein of unknown function		Intermediate and rare
SJC1043_03190		-0.857	2.04E-02	**-1.052**	**9.21E-03**	-0.892	6.78E-04	Type I secretion MFP, bacteriocin export		Intermediate and rare
SJC1043_03261		**2.495**	**6.62E-08**	**2.590**	**5.48E-15**	**1.670**	**7.02E-04**	Lytic polysaccharide monooxygenase	Lytic polysaccharide monooxygenase	Lineage specific intermediate
SJC1043_03279		**1.365**	**7.48E-05**	0.113	9.95E-01	**1.050**	**3.52E-03**	Protein of unknown function		Lineage specific intermediate
SJC1043_03289	tagC_B	0.972	3.40E-01	1.741	1.69E-02	**1.331**	**5.48E-03**	Type VI secretion system accessory protein	T6SS-2 (i3v2)	Core and intermediate
SJC1043_03291	tagAB_B	0.790	5.27E-01	1.575	2.33E-02	**1.306**	**7.92E-03**	Type VI secretion system accessory protein	T6SS-2 (i3v2)	Core and intermediate
SJC1043_03293	tssH_B	1.115	1.25E-01	0.972	1.66E-01	**1.139**	**9.38E-03**	Type VI secretion system ATPase	T6SS-2 (i3v2)	Intermediate and rare
SJC1043_03294	tssG_B	1.512	4.67E-02	1.252	6.23E-02	**1.518**	**3.09E-03**	Type VI secretion system baseplate protein	T6SS-2 (i3v2)	Intermediate and rare
SJC1043_03295	tssF_B	1.209	2.60E-01	1.339	7.78E-02	**1.581**	**2.29E-03**	Type VI secretion system baseplate protein	T6SS-2 (i3v2)	Intermediate and rare
SJC1043_03297	hcp_B	**1.545**	**5.95E-04**	**1.760**	**1.65E-04**	0.681	2.06E-01	Type VI secretion system tube protein	T6SS-2 (i3v2)	Core and intermediate
SJC1043_03299	tssB_B	**1.529**	**2.52E-04**	0.463	6.87E-01	**1.405**	**1.56E-04**	Type VI secretion system sheath protein	T6SS-2 (i3v2)	Core and intermediate
SJC1043_03300	tssA_B	**1.155**	**3.80E-03**	0.717	5.14E-01	0.886	2.97E-02	Type VI secretion system protein	T6SS-2 (i3v2)	Core, intermediate and rare
SJC1043_03371		0.677	7.36E-02	-0.242	9.85E-01	**1.042**	**4.21E-04**	Protein of unknown function		Intermediate and rare
SJC1043_03378		**1.637**	**1.70E-06**	**1.389**	**1.65E-04**	**1.220**	**6.99E-04**	ParB/sulfiredoxin-like	Putative antibiotic NRPS	Intermediate and rare
SJC1043_03379		**1.018**	**1.86E-03**	0.571	5.10E-02	0.658	5.04E-02	Non-ribosomal peptide synthetase	Putative antibiotic NRPS	Lineage specific intermediate
SJC1043_03380		**1.856**	**4.51E-10**	**1.782**	**5.44E-12**	**1.031**	**8.25E-03**	Non-ribosomal peptide synthetase	Putative antibiotic NRPS	Lineage specific intermediate
SJC1043_03381		**1.226**	**5.84E-07**	**1.026**	**4.81E-03**	0.728	1.87E-04	Similar to azoleucine resistance protein	Putative antibiotic NRPS	Lineage specific intermediate
SJC1043_03382		**2.777**	**8.54E-30**	**2.041**	**2.34E-08**	**1.772**	**1.33E-11**	NAD-dependent oxidoreductase (ornithine cyclodeaminase family)	Putative antibiotic NRPS	Lineage specific intermediate
SJC1043_03383		**2.389**	**2.26E-19**	**1.617**	**2.12E-04**	**1.392**	**1.61E-04**	Member of the cysteine synthase cystathionine beta-synthase family	Putative antibiotic NRPS	Intermediate and rare
SJC1043_03384		**1.112**	**2.53E-03**	**1.247**	**6.57E-04**	0.515	2.33E-01	Protein of unknown function		Core, intermediate and rare
SJC1043_03441		**1.105**	**7.80E-04**	0.255	9.68E-01	0.882	9.38E-03	Phage repressor protein C-like, contains Cro/C1-type HTH DNA-binding and peptidase S24 domains		Collection core
SJC1043_03476		**1.778**	**1.39E-03**	1.376	4.22E-02	0.848	3.34E-01	Protein of unknown function		Collection core
SJC1043_03489		0.193	9.12E-01	0.194	9.68E-01	**1.110**	**3.09E-03**	Protein of unknown function		Lineage specific intermediate
SJC1043_03519		1.096	1.06E-01	-1.581	1.25E-02	**1.330**	**6.40E-03**	Non-coding RNA		NA
SJC1043_03525		**2.171**	**1.20E-09**	**2.410**	**2.45E-08**	**1.662**	**9.87E-08**	Putative vitamin uptake transporter	Phosphodiesterase locus	Intermediate and rare

SJC1043_03526	cpdA_1	**3.294**	**8.91E-65**	**2.939**	**6.88E-32**	**2.301**	**1.95E-48**	3',5'-cyclic AMP phosphodiesterase CpdA	Phosphodiesterase locus	Intermediate and rare
SJC1043_03527		**1.627**	**4.87E-14**	**1.502**	**3.63E-08**	**1.227**	**9.62E-06**	Protein of unknown function	Phosphodiesterase locus	Intermediate and rare
SJC1043_03528		**1.939**	**5.30E-21**	**1.312**	**8.08E-10**	**1.613**	**1.47E-13**	Transcriptional regulator (Cro/C1-type HTH DNA-binding domain)	Phosphodiesterase locus	Intermediate and rare
SJC1043_03539	ompC	**1.239**	**1.57E-06**	**1.091**	**6.68E-03**	0.320	5.10E-01	Outer membrane porin		Collection core
SJC1043_03540		**1.468**	**6.66E-03**	0.784	5.33E-01	0.641	4.37E-01	Non-coding RNA (MicF)		NA
SJC1043_03664	ccmB	**-1.313**	**1.21E-06**	0.189	9.69E-01	-0.584	9.84E-02	Biogenesis of c-type cytochromes, heme exporter		Collection core
SJC1043_03688		**3.554**	**5.47E-03**	2.147	8.87E-01	1.264	6.62E-01	Protein of unknown function		Collection core
SJC1043_03725	crgA	**1.721**	**7.67E-08**	**2.053**	**9.60E-14**	**1.134**	**6.66E-05**	CrgA-like LysR-family transcriptional regulator	CrgA-like transcriptional regulator	Collection core
SJC1043_03729		**1.681**	**6.66E-03**	**1.605**	**2.11E-03**	1.117	2.27E-01	FlxA-like protein		Collection core
SJC1043_04027		**1.101**	**6.49E-03**	0.908	2.40E-01	0.435	5.16E-01	DoxX/SURF4-family membrane protein		Collection core
SJC1043_04040		**1.228**	**1.75E-03**	0.009	1.00E+00	**1.193**	**2.09E-03**	YqjD/ElaB-family inner membrane protein		Collection core
SJC1043_04252		**-1.181**	**1.30E-03**	**1.274**	**1.12E-04**	-0.842	8.27E-02	Protein of unknown function		Intermediate and rare
SJC1043_04280		**-1.501**	**1.85E-04**	-1.097	1.73E-01	-0.690	2.36E-01	Monosaccharide ABC transporter, permease component		Collection core
SJC1043_04284		**-2.052**	**2.91E-04**	-0.654	5.40E-01	-1.407	4.05E-02	DeoR/GlpR-family transcriptional repressor		Collection core
SJC1043_04449		**1.066**	**1.54E-04**	**1.012**	**1.53E-03**	0.194	7.86E-01	Transcriptional regulator (Cro/C1-type HTH DNA-binding domain)	Queuosine synthesis	Collection core
SJC1043_04450	yhhQ	**1.119**	**5.69E-05**	0.975	3.78E-07	0.381	2.01E-01	Queuosine precursor transporter	Queuosine synthesis	Collection core
SJC1043_04451	queF	**1.297**	**7.57E-07**	**1.450**	**3.58E-11**	0.535	7.30E-02	NADPH-dependent 7-cyano-7-deazaguanine reductase, QueF type 2	Queuosine synthesis	Collection core
SJC1043_04483*	cpdA_2	-0.789	8.60E-04	-0.470	2.10E-01	-0.985	3.98E-04	3',5'-cyclic AMP phosphodiesterase CpdA		Collection core
SJC1043_04667	csbD	**2.014**	**3.02E-06**	**1.452**	**1.81E-03**	**1.300**	**5.64E-03**	YjbJ/CsbD-family putative stress response protein	CsbD-like protein	Collection core
SJC1043_04793	yehU	**-1.128**	**6.61E-04**	-0.570	4.98E-01	-0.875	1.51E-02	Sensor histidine kinase (LytS/YehU-like)		Collection core
SJC1043_04925	iolG	-1.438	4.01E-02	-1.274	2.42E-01	**-1.669**	**6.62E-03**	Inositol 2-dehydrogenase		Collection core
SJC1043_04937	faeA	**-1.693**	**5.02E-08**	**-1.532**	**2.68E-10**	-0.908	4.65E-03	FaeA-family fimbrial protein transcriptional regulator	Fimbrial	Collection core
SJC1043_04938		**-2.884**	**8.51E-10**	**-2.439**	**4.56E-09**	-1.612	1.09E-02	Peptidyl-prolyl cis-trans isomerase	Fimbrial	Core and intermediate
SJC1043_04939		**-2.752**	**6.80E-09**	**-2.114**	**3.49E-11**	**-1.725**	**3.19E-03**	Protein of unknown function	Fimbrial	Core and intermediate
SJC1043_04940	smfA/fimA	**-3.652**	**1.51E-18**	**-3.008**	**1.50E-16**	**-2.876**	**1.57E-12**	Fimbrial protein (pilin)	Fimbrial	Collection core
SJC1043_04941	smfA/fimA	**-3.124**	**8.61E-07**	**-2.871**	**4.10E-13**	**-2.124**	**6.89E-03**	Fimbrial protein (pilin)	Fimbrial	Collection core
SJC1043_04942	papC	**-3.110**	**7.41E-11**	**-2.800**	**6.92E-18**	**-1.981**	**1.56E-04**	Fimbrial outer membrane usher protein	Fimbrial	Collection core
SJC1043_04943	papD	**-3.500**	**3.41E-18**	**-2.760**	**5.67E-23**	**-2.607**	**1.88E-09**	Fimbrial assembly chaperone protein	Fimbrial	Collection core
SJC1043_04944	smfA/fimA	**-3.157**	**3.18E-05**	**-2.721**	**2.09E-05**	-1.879	3.08E-02	Fimbrial protein (pilin)	Fimbrial	Collection core
SJC1043_04945	lpfD	**-3.103**	**7.41E-11**	**-2.533**	**9.06E-13**	**-2.108**	**8.84E-05**	Fimbrial protein (minor pilin/adhesin)	Fimbrial	Core and intermediate
SJC1043_04946	smfA/fimA	**-3.121**	**1.55E-06**	**-2.818**	**1.56E-06**	**-2.006**	**9.38E-03**	Fimbrial protein (pilin)	Fimbrial	Collection core
SJC1043_04947	fimW	**-2.702**	**6.11E-04**	**-2.623**	**1.35E-07**	-1.501	5.39E-02	Fimbriae biosynthesis transcriptional regulator	Fimbrial	Collection core
SJC1043_04964	uspA	-0.796	1.20E-01	0.461	5.26E-01	**-1.122**	**3.80E-05**	Universal stress protein A		Collection core
SJC1043_04980		**-2.613**	**3.72E-24**	**-2.412**	**2.02E-26**	**-1.767**	**3.24E-16**	BetR family transcriptional regulator	BetR	Collection core
SJC1043_04984		**1.054**	**5.82E-04**	0.553	2.30E-01	0.752	3.52E-02	TetR family transcriptional regulator		Collection core
SJC1043_04985		0.421	4.47E-01	**1.433**	**2.12E-04**	-0.571	3.73E-02	Protein of unknown function		Collection core
SJC1043_05048	glpF	**-1.490**	**9.30E-04**	-0.846	1.23E-01	**-1.504**	**1.19E-04**	Glycerol MIP channel protein	Glycerol uptake and metabolism	Collection core
SJC1043_05049	glpK	**-1.033**	**3.06E-04**	-0.627	8.28E-02	**-1.102**	**8.50E-06**	Glycerol kinase	Glycerol uptake and metabolism	Collection core

Table S2. Genes displaying significantly altered transcript levels between BetR system-intact and BetR system-disrupted strains according to RNAseq analysis. Related to Figure 5.
Genes are included if they showed a significant difference in transcript abundance (log2 fold change > 1 or < -1, adjusted p value < 0.01) in at least one of the three RNAseq comparisons between the strains indicated.
Green shading and bold text indicates significantly increased transcript level in the BetR/Reg2-intact strain compared with the BetR- or Reg2-disrupted strain in that comparison.
Blue shading and bold text indicates significantly decreased transcript level in the BetR/Reg2-intact strain compared with the BetR- or Reg2- disrupted strain in that comparison.
'Gene cluster/group' corresponds to categories shown in Figure 5 and 'Conservation' refers to pangenome category within *Serratia marcescens* as defined using Twilight (see Figure 5).
*Gene SJC1043_04483 just missed the fold change cut off for being considered significantly altered but is discussed in the main text therefore included here (grey box)

Table S3. Strains and Plasmids used in this study. Related to STAR Methods.

Name	Description/ genotype	Source / Reference
**Strains**		
Serratia marcescens		
Db10	Wild type (isolated from *Drosophila melanogaster*)	[S2]
SJC11	Db10 Δ*tssE* (Δ*SMDB11_2271*)	[S3]
RH02	Db10 BetR^{S42R} (*SMDB11_3959* with T126G single base pair mutation)	This study
SAN5	Db10 Δ*alb4-5* (Δ*SMDB11_2290-2289*)	[S4]
SJC1043	Wild type clinical isolate	[S5]
DJW1	SJC1043 Δ*tssE* (Δ*SJC1043_03158*), inactivation of T6SS_i3v1	This study
GM149	SJC1043 BetR^{1048} (intact *betR* gene, *SJC1043_04980*, replaced by disrupted version from SJC1048)	This study
GM162	SJC1043 BetR^{1051} (intact *betR* gene, *SJC1043_04980*, replaced by disrupted version from SJC1051)	This study
KM95	SJC1043 BetR^{1051} Δ*papD* (Δ*SJC1043_04943*)	This study
GM170	SJC1043 Reg2^{1070} (intact *reg2* gene, *SJC1043_04979*, replaced by disrupted version from SJC1070)	This study
GB12	SJC1043 Reg2^{1070} Δ*tssE*	This study
KM96	SJC1043 Δ*cpdA1* (Δ*SJC1043_03526*)	This study
KM93	SJC1043 Δ*cpdA2* (Δ*SJC1043_04483*)	This study
KM97	SJC1043 Δ*crgA* (Δ*SJC1043_03725*)	This study
KM99	SJC1043 Δ*csbD* (Δ*SJC1043_04667*)	This study
GB22	SJC1043 Δ*cpdA1* Δ*cpdA2*	This study
AH10	SJC1043 Δ*cpdA1* Δ*tssE*	This study
AH11	SJC1043 Δ*cpdA2* Δ*tssE*	This study
AH12	SJC1043 Δ*cpdA1* Δ*cpdA2* Δ*tssE*	This study
SJC1048	Wild type clinical isolate	[S5]
GM163	SJC1048 Δ*tssE*, inactivation of T6SS_i3v1	This study
GM148	SJC1048 BetR^{1043} (disrupted *betR* gene, *SJC1048_04981* Asp77fs, replaced by intact version from SJC1043)	This study
GM166	SJC1048 BetR^{1043} Δ*tssE*	This study
SJC1051	Wild type clinical isolate	[S5]
DJW5	SJC1051 Δ*tssE*, inactivation of T6SS_i3v1	This study
GM161	SJC1051 BetR^{1043} (disrupted *betR* gene, *SJC1051_05037* Gln53fs, replaced by intact version from SJC1043)	This study
GM167	SJC1051 BetR^{1043} Δ*tssE*	This study
KM94	SJC1051 Δ*papD* (Δ*SJC1051_05000*)	This study
SJC1070	Wild type clinical isolate	[S5]
GM165	SJC1070 Δ*tssE*, inactivation of T6SS_i3v1	This study
GM168	SJC1070 Reg2^{1043} (disrupted *reg2* gene, *SJC1070_01854* Leu288fs, replaced by intact version from SJC1043)	This study
GM169	SJC1070 Reg2^{1043} Δ*tssE*	This study
SJC1044	Wild type clinical isolate	[S5]
BL1	SJC1044 Δ*tssE*, inactivation of T6SS_i3v1	This study
SJC1045	Wild type clinical isolate	[S5]
DJW2	SJC1045 Δ*tssE*, inactivation of T6SS_i3v1	This study
SJC1046	Wild type clinical isolate	[S5]
DJW3	SJC1046 Δ*tssE*, inactivation of T6SS_i3v1	This study

SJC1050	Wild type clinical isolate	[S5]
DJW4	SJC1050 Δ*tssE*, inactivation of T6SS_i3v1	This study
SJC1052	Wild type clinical isolate	[S5]
GM164	SJC1052 Δ*tssE*, inactivation of T6SS_i3v1	This study
SJC1054	Wild type clinical isolate	[S5]
DJW6	SJC1054 Δ*tssE*, inactivation of T6SS_i3v1	This study
SJC1058	Wild type clinical isolate	[S5]
DJW7	SJC1058 Δ*tssE*, inactivation of T6SS_i3v1	This study
SJC1061	Wild type clinical isolate	[S5]
DJW8	SJC1061 Δ*tssE*, inactivation of T6SS_i3v1	This study
SJC1062	Wild type clinical isolate	[S5]
DJW9	SJC1062 Δ*tssE*, inactivation of T6SS_i3v1	This study
SJC1039	Wild type clinical isolate	[S5]
GB07	SJC1039 Δ*tssE* (Δ*SJC1039_01480*), inactivation of single T6SS (i3v1)	This study
KM92	SJC1039 BetR^{1042} (intact *betR* gene, *SJC1039_03124*, replaced by disrupted version from SJC1042)	This study
GB09	SJC1039 BetR^{1042} Δ*tssE*	This study
SJC1042	Wild type clinical isolate	[S5]
GB08	SJC1042 Δ*tssE*, inactivation of single T6SS (i3v1)	This study
KM98	SJC1042 BetR^{1039} (disrupted *betR* gene, *SJC1042_00416* Asp2fs, replaced by intact version from SJC1039)	This study
GB10	SJC1042 BetR^{1039} Δ*tssE*	This study
SM39	Wild type clinical isolate (intrinsically Sm^R)	[S2]
Escherichia coli		
BW25113 *lacA*::Kn	Deletion of *lacA* with insertion of Kn-resistance cassette in BW25113, retrieved from the Keio mutant library; Kn^R	[S6]
CC118λ*pir*	Cloning host and donor strain for pKNG101-derived allelic exchange plasmids (λ*pir*)	[S7]
HH26 pNJ5000	Mobilizing strain for conjugal transfer	[S8]
Pseudomonas fluorescens		
KT02	Sm^R derivative of *P. fluorescens* 55	[S3]
Bacillus subtilis		
NRS1473	NCIB3610 *sacA::*$P_{hy\text{-}spank}$*-gfpmut2 (kan)*	[S9]
**Plasmids**		
pKNG101	Suicide vector for allelic exchange (Sm^R, *sacBR, mobRK2, oriR6K*)	[S10]
pSC2606	pKNG101-derived allelic exchange plasmid for the generation of chromosomal in-frame Δ*tssE* deletion in T6SS_i3v1 in SJC1043-SJC1070	This study
pSC3705	pKNG101-derived allelic exchange plasmid for the generation of chromosomal in-frame Δ*tssE* deletion in SJC1039 and SJC1042	This study
pSC2565	pKNG101-derived allelic exchange plasmid for the introduction of the intact *betR* allele from SJC1043 (*SJC1043_04980*) to replace the disrupted *betR* alleles in SJC1048 and SJC1051	This study
pSC2566	pKNG101-derived allelic exchange plasmid for the introduction of the disrupted *betR* allele from SJC1048 (*SJC1048_04981* Asp77fs) to replace the intact *betR* allele in SJC1043	This study

pSC2567	pKNG101-derived allelic exchange plasmid for the introduction of the disrupted *betR* allele from SJC1051 (*SJC1051_05037* Gln53fs) to replace the intact *betR* allele in SJC1043	This study
pSC2576	pKNG101-derived allelic exchange plasmid for the introduction of the intact *reg2* allele from SJC1043 (*SJC1043_04979*) to replace the disrupted *reg2* allele in SJC1070	This study
pSC2577	pKNG101-derived allelic exchange plasmid for the introduction of the disrupted *reg2* allele from SJC1070 (*SJC1070_01854* Leu288fs) to replace the intact *reg2* allele in SJC1043	This study
pSC3305	pKNG101-derived allelic exchange plasmid for the reintroduction of the $BetR^{S42R}$ allele (*SMDB11_3959* with T126G single base pair mutation) in the chromosome of Db10	This study
pSC2360	pKNG101-derived allelic exchange plasmid for the introduction of the intact *betR* allele from SJC1039 (*SJC1039_03124*) to replace the disrupted *betR* allele in SJC1042	This study
pSC2361	pKNG101-derived allelic exchange plasmid for the introduction of the disrupted *betR* allele from SJC1042 (*SJC1042_00416* Asp2fs) to replace the intact *betR* allele in SJC1039	This study
pSC2362	pKNG101-derived allelic exchange plasmid for the generation of chromosomal in-frame Δ*csbD* deletion in SJC1043	This study
pSC2363	pKNG101-derived allelic exchange plasmid for the generation of chromosomal in-frame Δ*cpdA1* deletion in SJC1043	This study
pSC2364	pKNG101-derived allelic exchange plasmid for the generation of chromosomal in-frame Δ*cpdA2* deletion in SJC1043	This study
pSC2365	pKNG101-derived allelic exchange plasmid for the generation of chromosomal in-frame Δ*crgA* deletion in SJC1043	This study
pSC2366	pKNG101-derived allelic exchange plasmid for the generation of chromosomal in-frame Δ*papD* deletion in SJC1043 and SJC1051	This study

Table S4. Oligonucleotide primers used in this study. Related to STAR Methods.

Plasmid	Sequence of relevant primers (5'-3')[a]	Description
pSC2606	TATATCTAGACGACCACGCTGCTGCTGATG	Forward primer to clone upstream region of SJC1043_03158 in pKNG101 (*Xba*I)
	TATAAAGCTTATTCATGTTCAGGCGCTCGCGTCG	Reverse primer to clone upstream region of SJC1043_03158 in pKNG101 (*Hin*dIII)
	TATAAAGCTTCACTTCGATCTGAAAGACATAGGATAAGGC	Forward primer to clone downstream region of SJC1043_03158 in pKNG101 (*Hin*dIII)
	TATAGTCGACCTGCTGCGCCTGCATGTC	Reverse primer to clone downstream region of SJC1043_03158 in pKNG101 (*Sal*I)
pSC3705	TATATCTAGAGAGCTGCAACGCCAG	Forward primer to clone upstream region of SJC1039_01480 in pKNG101 (*Xba*I)
	ATATAAGCTTGTCGTTCATGGTCAGG	Reverse primer to clone upstream region of SJC1039_01480 in pKNG101 (*Hin*dIII)
	TATAAAGCTTCTGAAAGACATAGGGTAAGG	Forward primer to clone downstream region of SJC1039_01480 in pKNG101 (*Hin*dIII)
	ATATGGGCCCCAGGTAAAACATCAG	Reverse primer to clone downstream region of SJC1039_01480 in pKNG101 (*Apa*I)
pSC2565, pSC2566, pSC2567	TATATCTAGAAGTAGAAATAATCAAAACGACTGA	Forward primer to clone region containing SJC1043_04980 from SJC1043, SJC1048 or SJC1051 in pKNG101 (*Xba*I)
	TATAGGATCCTTCGACAACTACCTGATGAA	Reverse primer to clone region containing SJC1043_04980 from SJC1043, SJC1048 or SJC1051 in pKNG101 (*Bam*HI)
pSC2576, pSC2577	TATATCTAGACAACGACAATCTGCGGCTGC	Forward primer to clone mutation-containing region in SJC1043_04979 from SJC1043 or SJC1070 in pKNG101 (*Xba*I)
	TATAGGGCCCGTTGTCATCCACCACCAGG	Reverse primer to clone mutation-containing region in SJC1043_04979 from SJC1043 or SJC1070 in pKNG101 (*Apa*I)
pSC3305	TATATCTAGACAACGACAATCTGCGGCTGC	Forward primer to clone mutation-containing region in SMDB11_3959 in pKNG101 (*Apa*I)
	TATAGGGCCCGTTGTCATCCACCACCAGG	Reverse primer to clone mutation-containing region in SMDB11_3959 in pKNG101 (*Xba*I)
pSC2360, pSC2361	TATAGTCGACCCTGATGCGAATCGTCC	Forward primer to clone mutation-containing region in SJC1039_03124 from SJC1039 or SJC1042 in pKNG101 (*Sal*I)
	TATAGGGCCCCTGTCGCAGAACCATCAGG	Reverse primer to clone mutation-containing region in SJC1039_03124 from SJC1039 or SJC1042 in pKNG101 (*Apa*I)
pSC2362	TATAGGATCCGTTATTTGCTGGACGGCATG	Forward primer to clone upstream region of SJC1043_04667 in pKNG101 (*Bam*HI)
	TATAAAGCTTTTTATTCATCGTCTCTCCGTTAACC	Reverse primer to clone upstream region of SJC1043_04667 in pKNG101 (*Hin*dIII)
	TATAAAGCTTCGCTGGTAATTTGCAGGG	Forward primer to clone downstream region of SJC1043_04667 in pKNG101 (*Hin*dIII)
	TATAGGGCCCGCAACGGCTGGAAGTG	Reverse primer to clone downstream region of SJC1043_04667 in pKNG101 (*Apa*I)
pSC2363	TATAGGATCCGTTTCTTGATCTCCACATTGCTTAC	Forward primer to clone upstream region of SJC1043_03526 in pKNG101 (*Bam*HI)
	TATAAAGCTTTACTTTCATGCGAGACCCTG	Reverse primer to clone upstream region of SJC1043_03526 in pKNG101 (*Hin*dIII)
	TATAAAGCTTCCCTGGTTTATTTAATCAACGTC	Forward primer to clone downstream region of SJC1043_03526 in pKNG101 (*Hin*dIII)
	TATAGGGCCCGTCCTATAGCGATTATGCGATC	Reverse primer to clone downstream region of SJC1043_03526 in pKNG101 (*Apa*I)
pSC2364	TATAGGATCCGGGCGCGATCGATAAC	Forward primer to clone upstream region of SJC1043_04483 in pKNG101 (*Bam*HI)
	TATAAAGCTTGCTTTCCAAATGGTGTCCTTT	Reverse primer to clone upstream region of SJC1043_04483 in pKNG101 (*Hin*dIII)
	TATAAAGCTTATGGACTCGGATGGATACTGA	Forward primer to clone downstream region of SJC1043_04483 in pKNG101 (*Hin*dIII)
	TATAGGGCCCGTCCTATAGCGATTATGCGATC	Reverse primer to clone downstream region of SJC1043_04483 in pKNG101 (*Apa*I)

pSC2365	TATAGGATCCGATAGATTTCGCCCTCCCC	Forward primer to clone upstream region of SJC1043_03725 in pKNG101 (*Bam*HI)
	TATAAAGCTTGTCATTCATTTTTGCATTATATGC	Reverse primer to clone upstream region of SJC1043_03725 in pKNG101 (*Hin*dIII)
	TATAAAGCTTGGTCAACCGCCCAGC	Forward primer to clone downstream region of SJC1043_03725 in pKNG101 (*Hin*dIII)
	TATAGGGCCCCCTATCTGCGCCGACC	Reverse primer to clone downstream region of SJC1043_03725 in pKNG101 (*Apa*I)
pSC2366	TATAGGATCCGCACGGTATCGCGGC	Forward primer to clone upstream region of SJC1043_04943 in pKNG101 (*Bam*HI)
	TATAAAGCTTTTTGTTCATGTCATTATTTCTCAGC	Reverse primer to clone upstream region of SJC1043_04943 in pKNG101 (*Hin*dIII)
	TATAAAGCTTGAGAAAAAATAAGGAGGTCCAGC	Forward primer to clone downstream region of SJC1043_04943 in pKNG101 (*Hin*dIII)
	TATAGGGCCCGTCCTATAGCGATTATGCGATC	Reverse primer to clone downstream region of SJC1043_04943 in pKNG101 (*Apa*I)

[a]Incorporated restriction sites for cloning into the respective vector are underlined.

Table S5. Reference T6SSs used for assigning subtype to T6SSs identified in *Serratia*. List includes experimentally-characterised T6SSs and manually-curated predicted T6SSs. Related to Figure S6 and STAR Methods.

Accession	Start	End	T6SS subtype	Tip label	Strain
AJ320483	322	27706	i3	AJ320483_i3	*Salmonella enterica* subsp. *enterica* serovar Typhimurium
AY424360	3625	24399	i4b	AY424360_i4b	*Edwardsiella tarda* strain PPD130/91
FJ790776	1	32212	i1	FJ790776_i1	*Vibrio alginolyticus* strain EPJS T6SS1
FJ790777	1	31102	i5	FJ790777_i5	*Vibrio alginolyticus* strain EPJS T6SS2
JF766712	1	38426	i3	JF766712_i3	*Salmonella enterica* strain GIFU10007
JX436460	1	17012	i1	JX436460_i1	*Campylobacter jejuni* strain 108
KC432605	1	24537	i4b	KC432605_i4b	*Acinetobacter baumannii* strain M2
KF678349	1	29754	i2	KF678349_i2	*Escherichia coli* strain TW-XM
NC_002506	110871	145577	i1	NC_002506_i1	*Vibrio cholerae* O1 biovar El Tor str. N16961
NC_002516	1797958	1827821	i1	NC_002516_i1	*Pseudomonas aeruginosa* PAO1
NC_002516	83380	123495	i3	NC_002516_i3	*Pseudomonas aeruginosa* PAO1
NC_002516	2599893	2632231	i4a	NC_002516_i4a	*Pseudomonas aeruginosa* PAO1
NC_002927	825621	868006	i3	NC_002927_i3	*Bordetella bronchiseptica* RB50
NC_002947	2984253	3012550	i1	NC_002947_i1	*Pseudomonas putida* KT2440
NC_002947	4590927	4618219	i1	NC_002947_2_i1	*Pseudomonas putida* KT2440
NC_002947	3470069	3499780	i4b	NC_002947_i4b	*Pseudomonas putida* KT2440
NC_003063	1460756	1488734	i5	NC_003063_i5	*Agrobacterium* fabrum str. C58
NC_003143	526127	562682	i3	NC_003143_i3	Yersinia pestis CO92
NC_003296	926494	971950	i4b	NC_003296_i4b	*Ralstonia pseudosolanacearum* GMI1000 plasmid pGMI1000MP
NC_004129	6897920	6930515	i3	NC_004129_i3	*Pseudomonas protegens* Pf-5
NC_004431	3217751	3253963	i2	NC_004431_i2	*Escherichia coli* CFT073
NC_004547	3829218	3869582	i1	NC_004547_i1	*Pectobacterium atrosepticum* SCRI1043
NC_004578	6149242	6189243	i1	NC_004578_i1	*Pseudomonas syringae* pv. *tomato* str. DC3000
NC_004603	1486402	1526996	i1	NC_004603_i1	*Vibrio parahaemolyticus* RIMD 2210633
NC_004605	1074208	1108114	i5	NC_004605_i5	*Vibrio parahaemolyticus* RIMD 2210633
NC_005966	2633143	2661510	i4b	NC_005966_i4b	*Acinetobacter baylyi* ADP1
NC_006349	735641	772695	i1	NC_006349_i1	*Burkholderia mallei* ATCC 23344
NC_006351	2036034	2064669	i1	NC_006351_i1	*Burkholderia pseudomallei* K96243
NC_007005	5866174	5915475	i4b	NC_007005_i4b	*Pseudomonas syringae* pv. *syringae* B728a
NC_007650	996276	1025886	i1	NC_007650_i1	*Burkholderia thailandensis* E264
NC_007651	3396298	3416317	i4b	NC_007651_i4b	*Burkholderia thailandensis* E264
NC_008095	5996770	6055445	i1	NC_008095_i1	*Myxococcus xanthus* DK 1622
NC_008378	491368	527630	i5	NC_008378_i5	*Rhizobium johnstonii* 3841 plasmid pRL12
NC_008463	3806993	3840213	i1	NC_008463_i1	*Pseudomonas aeruginosa* UCBPP-PA14
NC_008463	82730	121410	i3	NC_008463_i3	*Pseudomonas aeruginosa* UCBPP-PA14
NC_008463	3011563	3044708	i4a	NC_008463_i4a	*Pseudomonas aeruginosa* UCBPP-PA14

NC_008570	1984799	2036268	i1	NC_008570_i1	*Aeromonas hydrophila* subsp. *hydrophila* ATCC 7966
NC_008702	4242264	4289066	i3	NC_008702_i3	*Azoarcus olearius*
NC_008752	1617808	1647773	i3	NC_008752_i3	*Paracidovorax citrulli* AAC00-1
NC_009085	1506250	1544065	i4b	NC_009085_i4b	*Acinetobacter baumannii* ATCC 17978
NC_009708	3850633	3874248	i3	NC_009708_i3	*Yersinia pseudotuberculosis* IP 31758
NC_009832	1958949	1988005	i2	NC_009832_i2	*Serratia proteamaculans* 568
NC_009832	3294549	3324584	i3	NC_009832_i3	*Serratia proteamaculans* 568
NC_010400	2061603	2081014	i4b	NC_010400_i4b	*Acinetobacter baumannii* SDF
NC_010465	3905709	3929297	i3	NC_010465_i3	*Yersinia pseudotuberculosis* YPIII
NC_010554	807789	834462	i1	NC_010554_i1	*Proteus mirabilis* HI4320
NC_011000	363015	382880	i4b	NC_011000_i4b	*Burkholderia cenocepacia* J2315
NC_011205	305501	331344	i3	NC_011205_i3	*Salmonella enterica* subsp. *enterica* serovar Dublin str. CT_02021853
NC_011274	1114451	1137704	i1	NC_011274_i1	*Salmonella enterica* subsp. *enterica* serovar Gallinarum str. 287/91
NC_012731	3190436	3214872	i2	NC_012731_i2	*Klebsiella pneumoniae* subsp. *pneumoniae* NTUH-K2044
NC_012779	2631752	2651730	i4b	NC_012779_i4b	*Edwardsiella ictaluri* 93-146
NC_013508	2555437	2575336	i4b	NC_013508_i4b	*Edwardsiella piscicida*
NC_013716	2914927	2946102	i3	NC_013716_i3	*Serratia proteamaculans* 568
NC_013716	3590954	3608523	i4a	NC_013716_i4a	*Serratia proteamaculans* 568
NC_013956	4583032	4612917	i2	NC_013956_i2	*Pantoea ananatis* LMG 20103
NC_013956	2617516	2646006	i3	NC_013956_i3	*Pantoea ananatis* LMG 20103
NC_013961	3291637	3325348	i2	NC_013961_i2	*Erwinia amylovora* CFBP1430
NC_013961	3102718	3135195	i3	NC_013961_i3	*Erwinia amylovora* CFBP1430
NC_014121	1551980	1588894	i3	NC_014121_i3	*Enterobacter cloacae* subsp. *cloacae* ATCC 13047
NC_016810	304656	332038	i3	NC_016810_i3	*Salmonella enterica* subsp. *enterica* serovar Typhimurium str. SL1344
NC_016856	305365	332747	i3	NC_016856_i3	*Salmonella enterica* subsp. *enterica* serovar Typhimurium str. 14028S
NC_017626	4852875	4885437	i2	NC_017626_i2	*Escherichia coli* 042
NC_017626	4893076	4921378	i4b	NC_017626_i4b	*Escherichia coli* 042
NC_020064	1749566	1796753	i2	NC_020064_i2	*Serratia* sp. FGI94
NC_020064	2918666	2954830	i3	NC_020064_i3	*Serratia* sp. FGI94
NC_020211	3235473	3273364	i3	NC_020211_i3	*Serratia marcescens* WW4
NC_020211	3387021	3410437	i3	NC_020211_2_i3	*Serratia marcescens* WW4
NC_022000	916585	943300	i1	NC_022000_i1	*Proteus mirabilis* BB2000
NZ_AAKJ02000013	12546	38192	i1	NZ_AAKJ02000013_i1	*Vibrio cholerae* V52 gcontig_1103206898976
AP013063	2515048	2552477	i3	SM39	*Serratia marcescens* SM39
HG326223	2365124	240267	i3	GCA_000513215.1_DB11_genomic	*Serratia marcescens* Db11
NC_003228	2346972	2370693	iii	YP_211657.1	*Bacteroides fragilis* NCTC 9343

Supplemental References

S1. Li, J., Yao, Y., Xu, H.H., Hao, L., Deng, Z., Rajakumar, K., and Ou, H.Y. (2015). SecReT6: a web-based resource for type VI secretion systems found in bacteria. Environ. Microbiol. *17*, 2196-2202. https://doi.org/10.1111/1462-2920.12794.

S2. Iguchi, A., Nagaya, Y., Pradel, E., Ooka, T., Ogura, Y., Katsura, K., Kurokawa, K., Oshima, K., Hattori, M., Parkhill, J., et al. (2014). Genome evolution and plasticity of *Serratia marcescens*, an important multidrug-resistant nosocomial pathogen. Genome Biol. Evol. *6*, 2096-2110. https://doi.org/10.1093/gbe/evu160.

S3. Murdoch, S.L., Trunk, K., English, G., Fritsch, M.J., Pourkarimi, E., and Coulthurst, S.J. (2011). The opportunistic pathogen *Serratia marcescens* utilizes type VI secretion to target bacterial competitors. J. Bacteriol. *193*, 6057-6069. https://doi.org/10.1128/JB.05671-11.

S4. Gerc, A.J., Song, L., Challis, G.L., Stanley-Wall, N.R., and Coulthurst, S.J. (2012). The insect pathogen *Serratia marcescens* Db10 uses a hybrid non-ribosomal peptide synthetase-polyketide synthase to produce the antibiotic althiomycin. PloS One *7*, e44673. https://doi.org/10.1371/journal.pone.0044673.

S5. Williams, D.J., Grimont, P.A.D., Cazares, A., Grimont, F., Ageron, E., Pettigrew, K.A., Cazares, D., Njamkepo, E., Weill, F.X., Heinz, E., et al. (2022). The genus *Serratia* revisited by genomics. Nat. Commun. *13*, 5195. https://doi.org/10.1038/s41467-022-32929-2.

S6. Baba, T., Ara, T., Hasegawa, M., Takai, Y., Okumura, Y., Baba, M., Datsenko, K.A., Tomita, M., Wanner, B.L., and Mori, H. (2006). Construction of *Escherichia coli* K-12 in-frame, single-gene knockout mutants: the Keio collection. Mol. Syst. Biol. *2*, 2006 0008. https://doi.org/10.1038/msb4100050.

S7. Herrero, M., de Lorenzo, V., and Timmis, K.N. (1990). Transposon vectors containing non-antibiotic resistance selection markers for cloning and stable chromosomal insertion of foreign genes in gram-negative bacteria. J. Bacteriol. *172*, 6557-6567. https://doi.org/10.1128/jb.172.11.6557-6567.1990

S8. Grinter, N.J. (1983). A broad-host-range cloning vector transposable to various replicons. Gene *21*, 133-143. https://doi.org/10.1016/0378-1119(83)90155-5.

S9. Hobley, L., Ostrowski, A., Rao, F.V., Bromley, K.M., Porter, M., Prescott, A.R., MacPhee, C.E., van Aalten, D.M., and Stanley-Wall, N.R. (2013). BslA is a self-assembling bacterial hydrophobin that coats the *Bacillus subtilis* biofilm. Proc. Natl. Acad. Sci. U S A *110*, 13600-13605. https://doi.org/10.1073/pnas.1306390110.

S10. Kaniga, K., Delor, I., and Cornelis, G.R. (1991). A wide-host-range suicide vector for improving reverse genetics in Gram-negative bacteria: inactivation of the *blaA* gene of *Yersinia enterocolitica*. Gene *109*, 137-141. https://doi.org/10.1016/0378-1119(91)90599-7.
